# Supplementary material for: The Cryoelectron Microscopy Structure of the Type 1 Chaperone-Usher Pilus Rod
Source: Structure. 2017 Dec 5;25(12):1829–1838.e4. doi: 10.1016/j.str.2017.10.004 (PMC5719983; doi:10.1016/j.str.2017.10.004)
Supplement: Document S1. Figures S1–S4 and Table S1 [file mmc1.pdf]

Structure, Volume *25*

Supplemental Information

The Cryoelectron Microscopy Structure of the Type 1 Chaperone-Usher Pilus Rod

Manuela K. Hospenthal, Dawid Zyla, Tiago R.D. Costa, Adam Redzej, Christoph Giese, James Lillington, Rudi Glockshuber, and Gabriel Waksman

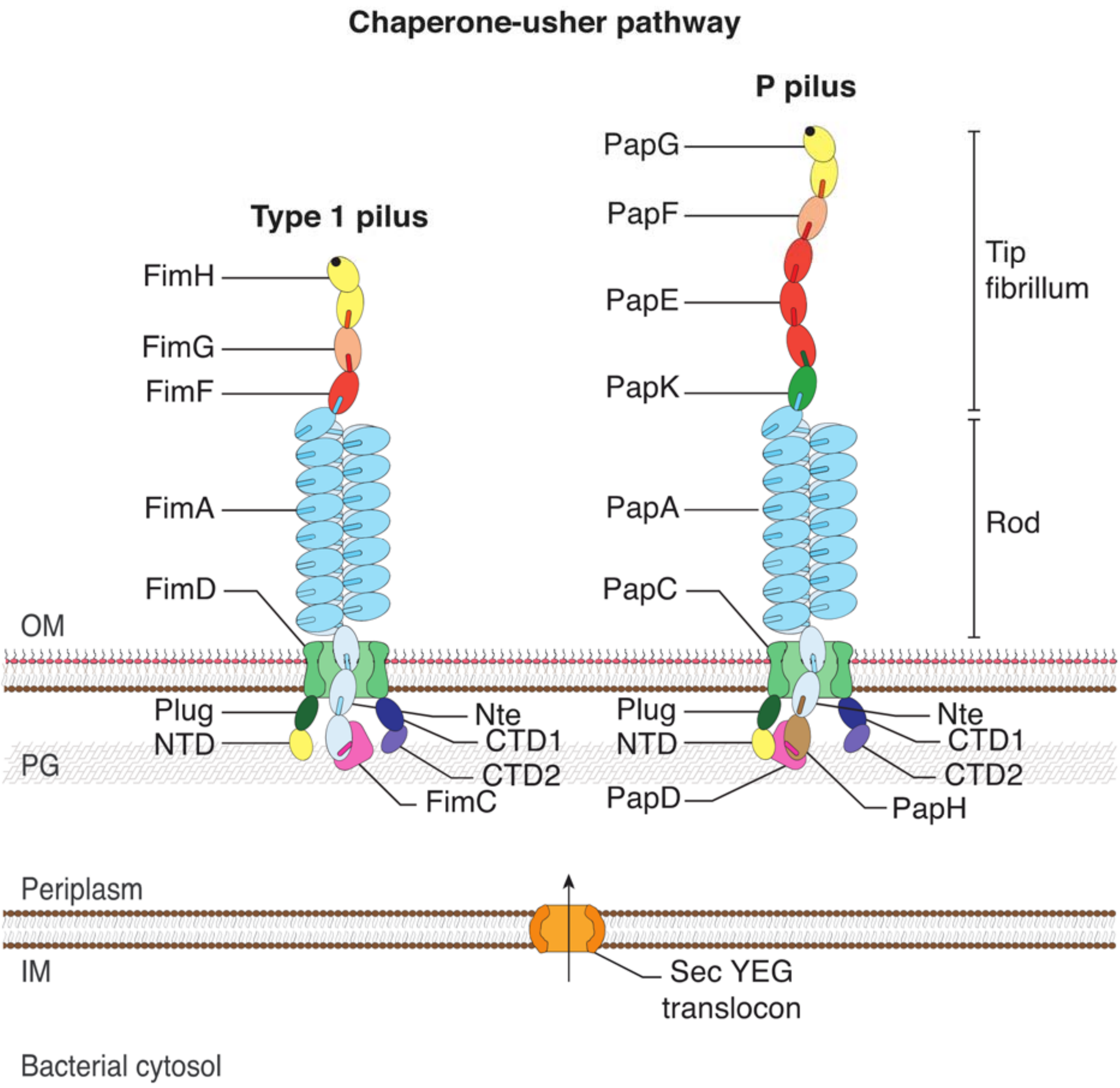
Chaperone-usher pathway
P pilus
Type 1 pilus
FimH
FimG
FimF
FimA
FimD
OM
Plug
NTD
PG
Nte
CTD1
CTD2
FimC
PapG
PapF
PapE
PapK
PapA
PapC
Plug
NTD
PapD
Nte
CTD1
CTD2
PapH
Tip
fibrillum
Rod
Periplasm
IM
Sec YEG
translocon
Bacterial cytosol

Figure S1: The Chaperone-Usher Pathway. Related to Figure 1, 2, 3, 4 and 5.

Pilin subunits are transported across the bacterial inner membrane (IM) via the Sec YEG translocon where they are received by a dedicated periplasmic chaperone (FimC, type 1 pili; PapD, P pili). The chaperone helps to fold and stabilise the pilin subunits and shuttles them to the outer membrane (OM)-embedded usher, where they are polymerised into a growing pilus. The usher is composed of a 24-stranded β-barrel pore, a periplasmic N-terminal domain (NTD), two periplasmic C-terminal domains (CTD1 and CTD2) and a plug domain. Once assembled, chaperone-usher pili are organised into two subassemblies: the tip fibrillum and a 1-2 μm long rod. The tip fibrillum is assembled first and requires the sequential addition of a single subunit of FimH, FimG and FimF (type 1 pili) or one subunit of PapG and PapF, 5-10 subunits of PapE and one PapK subunit (P pili). FimH and PapG are the adhesins composed of a C-terminal pilin domain and an N-terminal lectin domain, which is responsible for host-cell receptor interaction. The rod section is composed of ~1000 subunits of FimA (type 1 pili) or PapA (P pili).

a

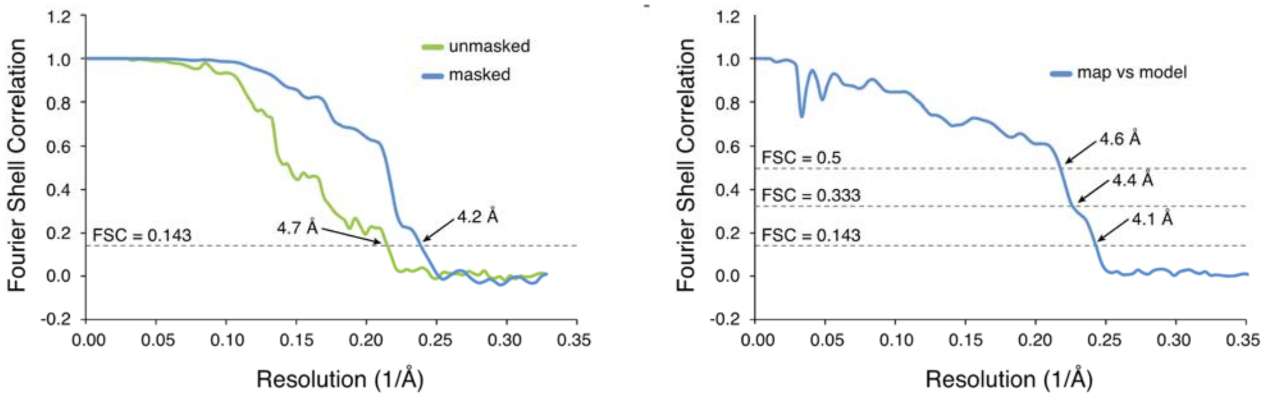

b

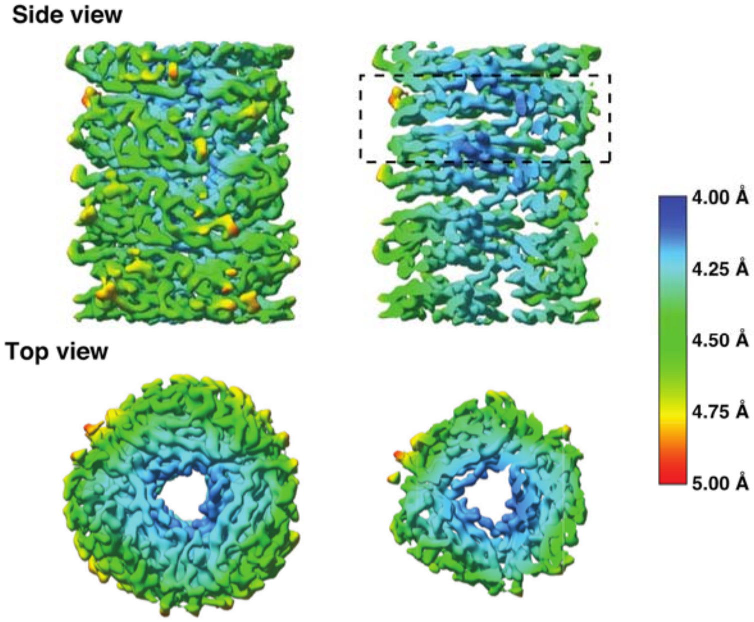

c

Molprobity score	1.12
All-atom clashscore	3.32
Ramachandran plot:	
Outliers (%)	0.00
Allowed (%)	1.93
Favoured (%)	98.07
RMS deviations (angles)(°)	0.62
RMS deviations (bonds)(Å)	0.00
Bad rotamers (%)	0.00

Figure S2: Resolution Analysis, Model Building and Refinement. Related to Figure 1.

a Left, Fourier Shell Correlation (FSC) curves calculated for unmasked (green) and masked (blue) reconstructions. Right, FSC curve calculated for the map versus model. Resolutions at the indicated FSC cut-offs are indicated on the graph. **b** Analysis of local resolution indicating that the luminal side of the pilus is better resolved than the exterior. A side view (upper panels) and bottom view (lower panels) of the map surface coloured according to the local resolution (indicated by the scale bar). The left panels show the whole map, whereas the right panels show a sliced view revealing more detail in the pilus interior. The dashed box on the sliced side view (upper right) represents the portion of the map included in the sliced top view (lower right). **c** Model validation statistics.

a

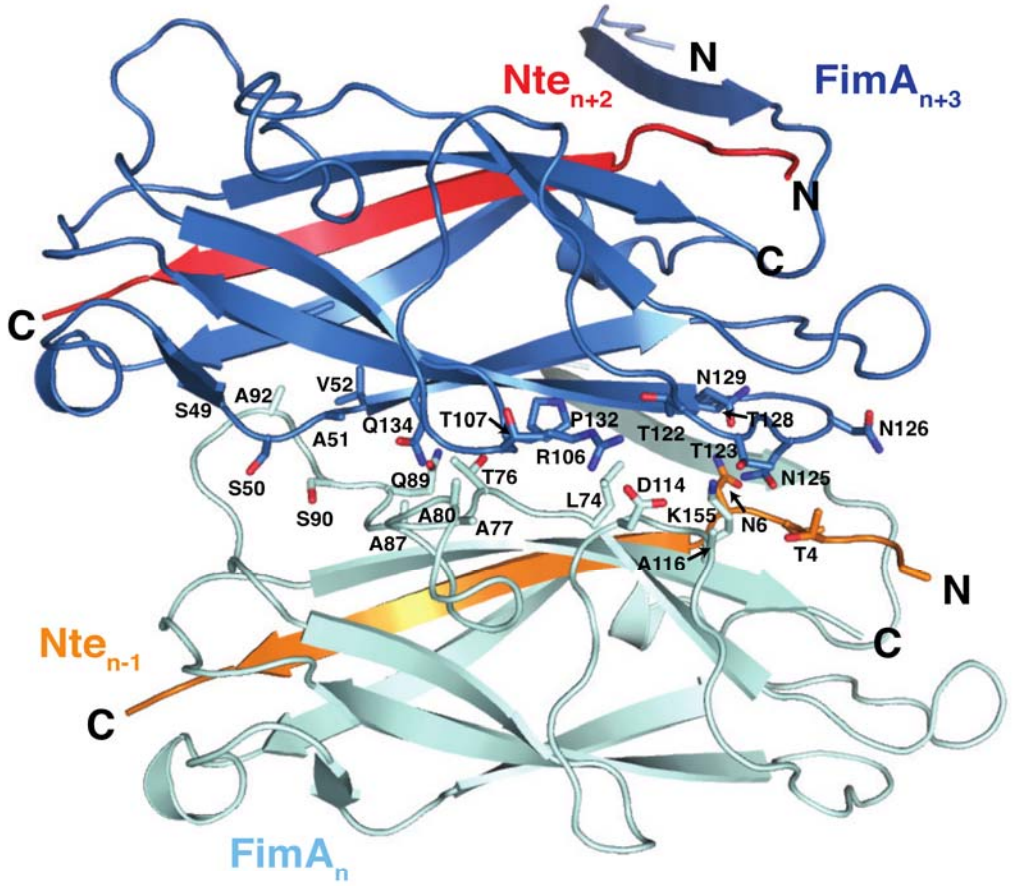

b

Stacking interface analysis

	Type 1	Type 1 (- DS)	P	P (- DS)
**Total buried surface area (Å)**	1616.2	1430.2	1453.0	1150.3

Figure S3: The Main Stacking Interface. Related to Figure 2.

a Zoomed-in view of the stacking interface in the same orientation as in Figure 2c showing participating side chains. The two FimA subunits are coloured in light and dark blue and are shown in ribbon representation. Donor-strands are coloured orange and red. Side chains are shown in stick representation with oxygens coloured red and nitrogens coloured blue. **b** Table displaying the total buried surface areas created by the n and n+3 (stacking) interface in type 1 and P pili. Values were calculated for the stacking interface with and without the donor strand (DS).

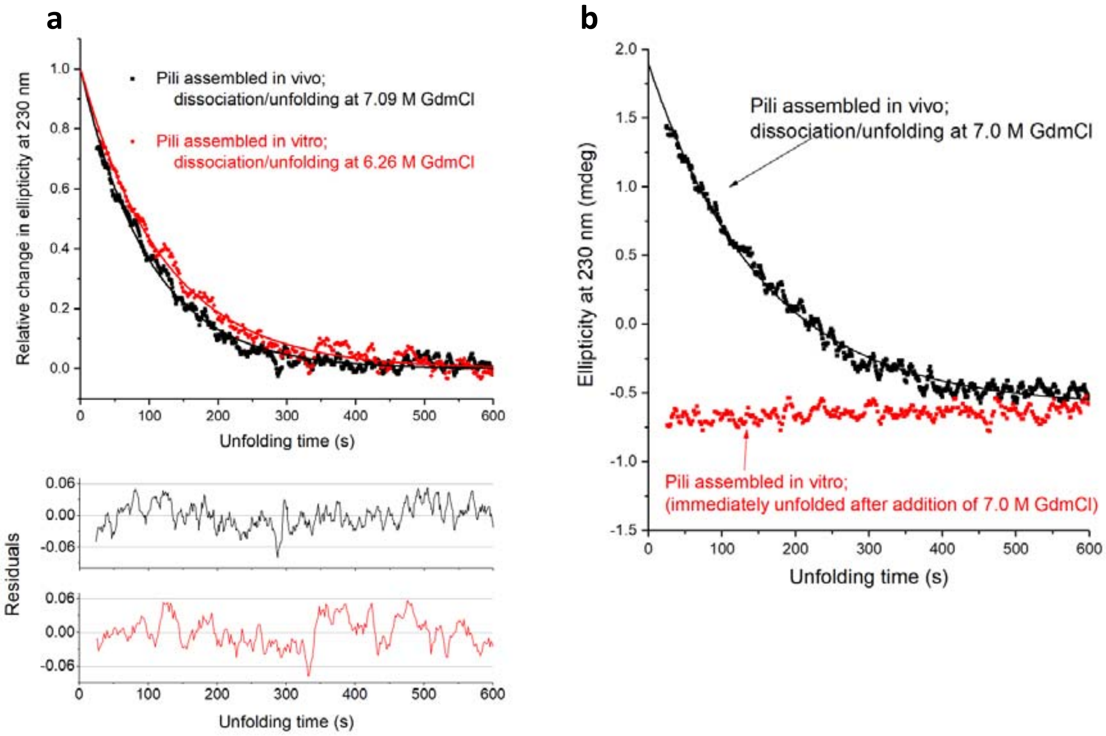

Figure S4: Unfolding/Dissociation Traces of Type 1 Pilus Rods Assembled *in vivo* and *in vitro.* Related to Figure 5.

a Unfolding traces recorded of type 1 pilus rods assembled *in vivo* (black symbols) and *in vitro* (red symbols) via the decrease in the far-UV CD signal at 230 nm at pH 2.1 and 25°C of pili assembled *in vivo* at 7.09 M GdmCl and pili assembled from FimA monomers *in vitro* at 6.26 M GdmCl. The data were fitted to a single first-order reaction (solid lines). The residuals at the bottom show that there are no systematic deviations form first-order kinetics. **b** Dissociation/unfolding at pH 2.1 and 7.0 M GdmCl. While pili assembled *in vivo* slowly dissociate with a half-life of 107±2 s, pili assembled *in vitro* already dissociate and unfold within the dead time of manual mixing under these conditions.

SUPPLEMENTAL TABLES

Table S1 Overview of the Refinement of Helical Parameters during EM Processing. Related to Experimental Procedures.

	**Twist (°)**	**Rise (Å)**
**After 3D classification**		
Class 1	115.202	8.02695
Class 2	114.999	8.00031
Class 3	114.984	8.01445
**After 3D refinement**		
Half 1	114.993	8.01008
Half 2	114.991	8.01367
Average	114.992	8.01188
