# Supplementary material for: The Cryoelectron Microscopy Structure of the Type 1 Chaperone-Usher Pilus Rod
Source: Structure. 2017 Dec 5;25(12):1829–1838.e4. doi: 10.1016/j.str.2017.10.004 (PMC5719983; doi:10.1016/j.str.2017.10.004)
Supplement: Document S2. Article plus Supplemental Information [file mmc2.pdf]

Structure

Article

The Cryoelectron Microscopy Structure of the Type 1 Chaperone-Usher Pilus Rod

Graphical Abstract

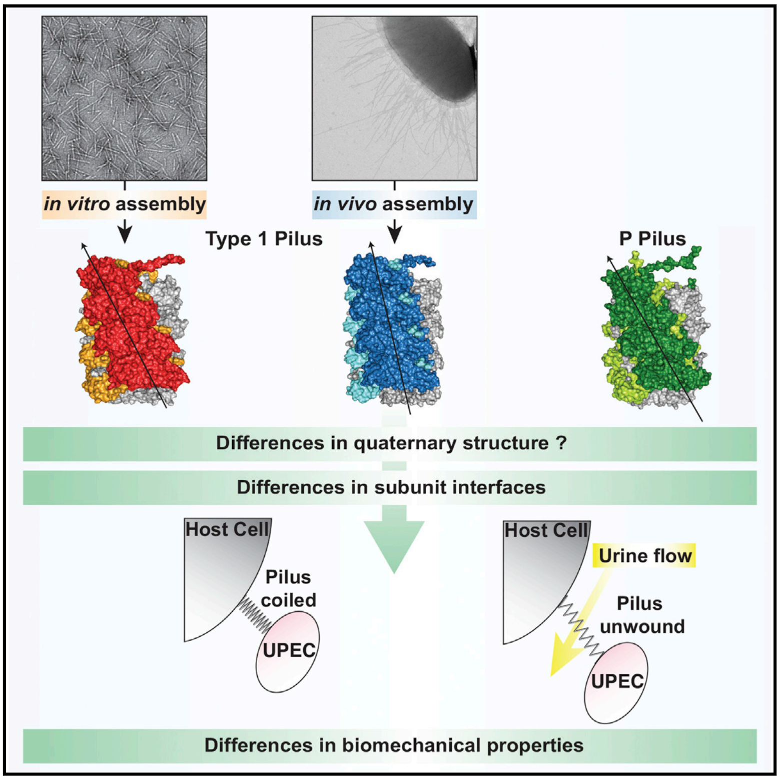

Highlights

- The atomic structure of the type 1 chaperone-usher pilus rod was solved by cryo-EM
- A comparison to the P pilus rod structure reveals similarities and key differences
- Our natively purified FimA rod structure differs to an *in vitro* assembled rod structure
- Unfolding kinetics suggest different quaternary assemblies are possible for FimA rods

Authors

Manuela K. Hospenthal, Dawid Zyla, Tiago R.D. Costa, ..., James Lillington, Rudi Glockshuber, Gabriel Waksman

Correspondence

g.waksman@mail.cryst.bbk.ac.uk

In Brief

Chaperone-usher pili are crucial virulence factors of uropathogenic *E. coli* that mediate host recognition and adhesion. Hospenthal et al. present the cryo-EM structure of the type 1 pilus rod explaining its "spring-like" biomechanical properties. Biochemical data suggest that the rod can exist in more than one helical quaternary assembly.

Hospenthal et al., 2017, Structure *25*, 1829–1838
December 5, 2017 © 2017 The Author(s). Published by Elsevier Ltd.
https://doi.org/10.1016/j.str.2017.10.004

CellPress

The Cryoelectron Microscopy Structure of the Type 1 Chaperone-Usher Pilus Rod

Manuela K. Hospenthal,[1] Dawid Zyla,[2] Tiago R.D. Costa,[1,3] Adam Redzej,[1,3] Christoph Giese,[2] James Lillington,[1] Rudi Glockshuber,[2] and Gabriel Waksman[1,4,*]
[1]Institute of Structural and Molecular Biology, University College London and Birkbeck, Malet Street, London WC1E 7HX, UK
[2]Institute of Molecular Biology and Biophysics, Swiss Federal Institute of Technology Zurich, Otto-Stern-Weg 5, 8093 Zurich, Switzerland
[3]These authors contributed equally
[4]Lead Contact
*Correspondence: g.waksman@mail.cryst.bbk.ac.uk
https://doi.org/10.1016/j.str.2017.10.004

SUMMARY

Adhesive chaperone-usher pili are long, supramolecular protein fibers displayed on the surface of many bacterial pathogens. The type 1 and P pili of uropathogenic *Escherichia coli* (UPEC) play important roles during urinary tract colonization, mediating attachment to the bladder and kidney, respectively. The biomechanical properties of the helical pilus rods allow them to reversibly uncoil in response to flow-induced forces, allowing UPEC to retain a foothold in the unique and hostile environment of the urinary tract. Here we provide the 4.2-Å resolution cryo-EM structure of the type 1 pilus rod, which together with the previous P pilus rod structure rationalizes the remarkable "spring-like" properties of chaperone-usher pili. The cryo-EM structure of the type 1 pilus rod differs in its helical parameters from the structure determined previously by a hybrid approach. We provide evidence that these structural differences originate from different quaternary structures of pili assembled *in vivo* and *in vitro*.

INTRODUCTION

Chaperone-usher pili are long, thin surface appendages displayed by many pathogenic Gram-negative bacteria (Thanassi et al., 1998). They serve to mediate important processes such as bacterial attachment to host tissues and biofilm formation, making them key virulence factors (Hospenthal et al., 2017; Thanassi et al., 2012). The two archetypal chaperone-usher pili are the type 1 and P pili of uropathogenic *Escherichia coli* (UPEC). UPEC are responsible for ~80% of community-acquired urinary tract infections (UTIs), and the role played by chaperone-usher pili in UTIs is firmly established (Flores-Mireles et al., 2015; McLellan and Hunstad, 2016).

The architecture of type 1 and P pili consists of a long, helically wound rod and a thin, flexible tip fibrillum located at the pilus' distal end (Sauer et al., 2004) (Figure S1). The subunit located at the very tip of the fibrillum is the adhesin (FimH for type 1 and PapG for P pili). The adhesin consists of an N-terminal lectin domain responsible for host cell receptor interaction and a C-terminal pilin domain that links to the next subunit in assembly (Choudhury et al., 1999; Dodson et al., 2001). The type I pilus adhesin FimH targets mannosylated host receptors such as the uroplakins of the bladder, whereas the P pilus adhesin PapG targets galabiose-containing glycosphingolipids, which are primarily expressed on the kidney epithelium (Hannan et al., 2012; Mulvey et al., 1998; Roberts et al., 1994). Thus, differential regulation of type 1 and P pilus expression may provide the basis of the observed tropism of UPEC for the bladder and the kidneys during a UTI (Spaulding and Hultgren, 2016).

Two additional subunits, present as single copies, FimG and FimF (in that order; Figure S1), complete the structure of the type 1 pilus tip fibrillum. The P pilus tip fibrillum is slightly longer and is completed by one subunit of PapF, 5–10 subunits of PapE, and one molecule of the adaptor subunit PapK. FimA (type 1 pilus) and PapA (P pilus) assemble into a ~1,000-subunit long helically coiled quaternary structure of ~3–4 subunits per turn known as the rod (Habenstein et al., 2015; Hospenthal et al., 2017, 2016) (Figure S1).

For the assembly of chaperone-usher pili, all pilus subunits or "pilins" are first transported into the periplasm via the Sec YEG translocon (Stathopoulos et al., 2000) (Figure S1). There, they are folded and stabilized by a dedicated periplasmic chaperone (Crespo et al., 2012; Vetsch et al., 2004), which shuttles each subunit to an outer membrane-embedded nanomachine termed the usher. On their own, pilins only show marginal thermodynamic stability and are unstable against aggregation and degradation because they consist of an incomplete immunoglobulin (Ig)-like fold lacking the seventh β strand, which exposes a hydrophobic groove on their surface (Barnhart et al., 2000; Choudhury et al., 1999; Sauer et al., 1999; Vetsch et al., 2004). This groove can be complemented by a donor strand originating either from the periplasmic chaperone in a process termed donor-strand complementation (DSC), or from the next subunit in assembly in a process termed donor-strand exchange (DSE) (Nishiyama et al., 2008; Sauer et al., 2002; Zavialov et al., 2003). A region consisting of 10–20 N-terminal residues known as the N-terminal extension (Nte), present on all subunits except for the adhesin, serves as the donor strand during DSE (Sauer et al., 2002; Waksman and Hultgren, 2009; Zavialov et al., 2003).

The usher receives chaperone-subunit complexes, catalyzes their assembly on the periplasmic side of the outer membrane (Nishiyama et al., 2008), and mediates the translocation of linear

This is an open access article under the CC BY license (http://creativecommons.org/licenses/by/4.0/).

chains of assembled subunits to the extracellular space (Geibel et al., 2013; Hospenthal et al., 2017; Phan et al., 2011; Remaut et al., 2008). The cycle of subunit incorporation by the usher is well characterized (reviewed in Waksman, 2017): all chaperone-subunit complexes are first recruited to the N-terminal domain (NTD) of the usher and subsequently transferred to two C-terminal domains (CTDs) that form a secondary chaperone-subunit binding platform. DSE occurs when a subunit located at the NTD reacts with the previously assembled subunit located at the CTDs. Indeed, the subunit at the NTD is positioned relative to the subunit at the CTDs in such a way that its Nte is close to the groove of the CTD-bound subunit and thus can "zip-in" into that groove, thereby displacing the chaperone and forming a native Nte-groove subunit-subunit interaction. At this point, the NTD-bound chaperone-subunit complex transfers to the CTDs with a rotation-and-translation motion that results in the extrusion of the pilus, one subunit at a time.

Type 1 and P pilus rods exhibit remarkable biomechanical properties enabling UPEC to resist being flushed out of the urinary tract during a UTI. In response to urine flow-induced forces, the helically coiled rod section of chaperone-usher pili can reversibly uncoil, thereby dissipating and relieving the force experienced by the adhesin-receptor complexes (Forero et al., 2006; Miller et al., 2006; Zakrisson et al., 2012). Several studies applying techniques such as atomic force microscopy (AFM) and optical tweezers have carefully deciphered and unpicked the processes that occur during the unwinding of the helical pilus rod (Andersson et al., 2008, 2006a, 2006b, 2006c, 2007; Fällman et al., 2005; Forero et al., 2006; Jass et al., 2004; Lugmaier et al., 2007; Miller et al., 2006; Zakrisson et al., 2012, 2013), and a mathematical model of the force versus elongation behavior of pili was developed (Andersson et al., 2006a; Jass et al., 2004). Despite their similarities, type 1 and P pilus rods behave differently in response to external forces. Notably, type 1 pili can respond faster to external force by entering a dynamic regime of elongation at lower elongation rates compared with P pili (6 nm/s versus 400 nm/s) (Andersson et al., 2007). In addition, type 1 pili require larger forces to peel apart the individual stack-to-stack interactions in the dynamic elongation mode (Andersson et al., 2008, 2007). It has been suggested that these differences allow type 1 pili to withstand the faster and more turbulent flows of the lower urinary tract (bladder), whereas P pili are biomechanically evolved to allow colonization of the upper urinary tract (kidney) (Andersson et al., 2008, 2007).

The previously reported structure of the P pilus rod provided the molecular explanation of how the main stacking interface in the rod (formed by every n and n+3 subunit) can break apart, while the much stronger DSE forces holding adjacent subunits together remain intact (Hospenthal et al., 2016). Here we present the 4.2-Å resolution cryoelectron microscopy (cryo-EM) structure of the related type 1 pilus rod. The comparison of the type 1 and P pilus rod structures begins to explain some of the differences in their biomechanical properties at the molecular level.

RESULTS

Structure Determination and Architecture of Type 1 Pilus Rods

Type 1 pili were expressed and assembled on the *Escherichia coli* cell surface, from which they were sheared and purified by density gradient centrifugation as described in STAR Methods. The purified sample (Figure 1A) was applied to grids and vitrified for cryo-EM analysis (Figure 1B). The resulting electron density map was resolved to an overall resolution of 4.2 Å (Figure S2A), which is consistent with the map showing clearly separated strands and visible density for bulky side chains (Figures 1C and 1D). An analysis of the local resolution of the EM map shows that the interior of the pilus rod is better resolved than the exterior (Figure S2B), and the lowest resolution is observed in outward-facing loops (including residues 66–67, 93–95, 108–109, 116–117, and 140–143), suggesting some disorder in these regions. Nevertheless, a near-atomic resolution model of the fully assembled type 1 pilus rod was built by fitting a previously determined nuclear magnetic resonance (NMR) solution structure of FimA (Puorger et al., 2011) into the EM map, followed by manual building and refinement (Figure S2C).

The pilus rod is ~72 Å in diameter and contains a hollow central lumen, which is ~14 Å wide (Figure 2A). Chaperone-usher pili do not have any known roles in secretion, despite the presence of a central hollow channel in both type 1 and the related P pili. Overall, the type 1 pilus is a right-handed superhelical assembly comprising 3.13 subunits per turn with an axial rise of 8.0 Å per subunit and a pitch of 25 Å (Figure 2B). An extensive subunit-subunit interaction network maintains the integrity of the superhelical quaternary structure, where each subunit interacts with a total of eight other subunits, four preceding and four succeeding subunits (n interacts with +1, +2, +3, +4 and −1, −2, −3, −4). This is similar to what has been observed for the P pilus structure, where each subunit interacts with a total of ten subunits (n also interacts with +5 and −5) (Hospenthal et al., 2016) (see below). By far the largest contribution to the subunit-subunit interaction network is made by the main stacking interface between every n and n+3 subunit pair (Figures 2C and S3). The majority of this interface is formed by residues on the loops linking β strands C and D (βC-βD loop) and D and E (βD-βE loop) (Figure 2C). The total buried surface area created by the interaction of the donor-strand complemented n and n+3 pilins is 1,616.2 $Å^2$ (or 1,430.2 $Å^2$ when the contribution of the donor strand is not taken into account) (Figure S3B).

FimA before and after Assembly into Type 1 Pili

Several structures of FimA have been determined before their assembly into pili. NMR was used to determine the structure of FimA in isolation where the hydrophobic groove was either self-complemented by FimA's own Nte (FimA, PDB: 2M5G; Walczak et al., 2014) or by a copy of the FimA Nte peptide, which was fused to the C terminus of the construct (FimAa, PDB: 2JTY; Puorger et al., 2011). The two structures differ in the orientation of donor-strand insertion with respect to the last β strand of the Ig-like pilin fold. In FimAa (PDB: 2JTY), the donor strand is inserted in a stable antiparallel fashion, identical to the orientation and register observed in the quaternary structure of the pilus; whereas in FimA (PDB: 2M5G) the donor strand is inserted in a less stable parallel arrangement (Figure 2D). In addition, two structures of FimA in complex with the chaperone FimC were determined by X-ray crystallography (Crespo et al., 2012), but only the highest-resolution

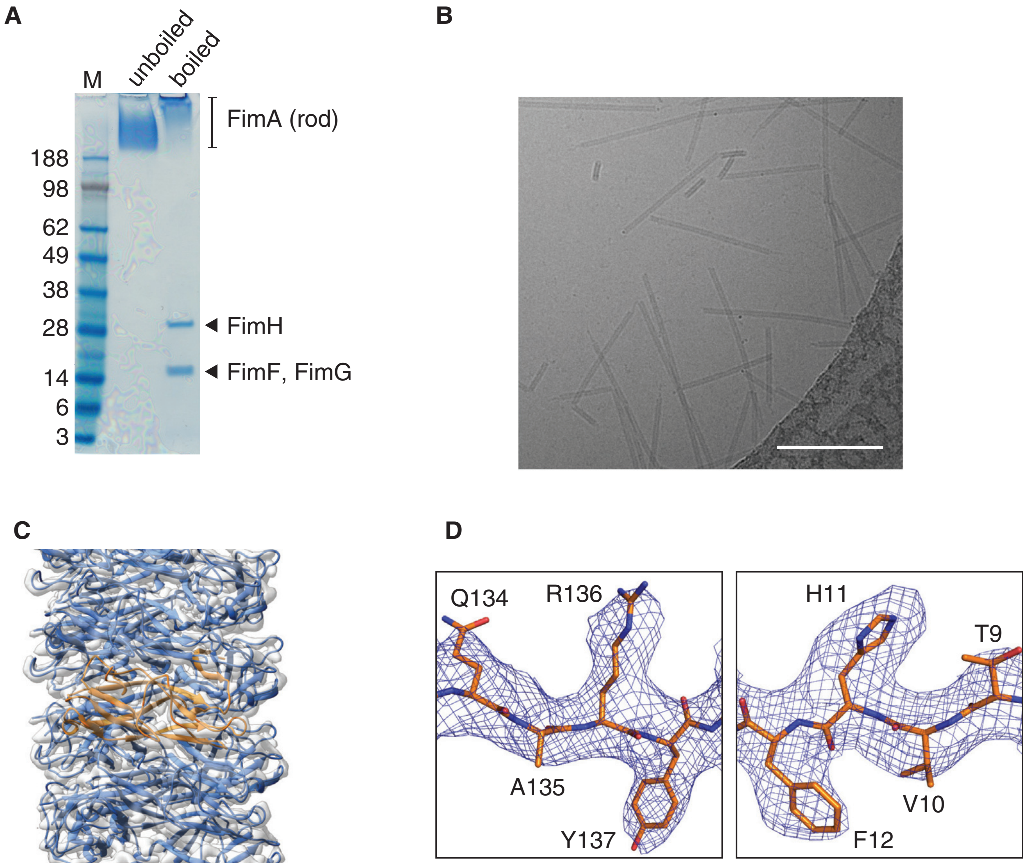

Figure 1. Purification and Cryo-EM Analysis of Type 1 Pili

(A) SDS-PAGE analysis of type 1 pili purified from the *E. coli* cell surface. When the sample was boiled for 15 min in the presence of SDS and 4.5 M urea, the pili partially dissociated and bands representing the monomers of some type 1 pilins were resolved. Mass spectrometry (LC-MS/MS) was performed to confirm the identity of all the bands (labeled).

(B) Representative electron micrograph of type 1 pili. Scale bar represents 100 nm.

(C) The refined type 1 pilus rod model is shown in ribbon representation in the experimentally derived electron density map (transparent gray surface). FimA subunits are colored blue, with the central FimA subunit highlighted in orange. For illustrative purposes, a model containing 12 molecules of FimA was created in PyMOL and a longer EM volume was created by imposing the helical parameters in real space.

(D) Regions of electron density around bulky side chains. Electron density is shown as a blue mesh and the model is shown in stick representation with carbons colored orange, oxygens red, and nitrogens blue. Residues are clearly labeled.

Please refer to Figure S1 for the general architecture of UPEC chaperone-usher pili and Figure S2 for details on resolution estimation, local resolution, and model validation.

structure (2.5 Å) (PDB: 4DWH) will be compared (Figure 2E). All three previous FimA structures were aligned to the structure of FimA assembled into a pilus using the pairwise alignment function of the DALI server (Holm and Laakso, 2016) (Figures 2D and 2E). The two solution structures of FimA align with a root-mean-square deviation (RMSD) (Cα) of 1.3 Å (FimA, PDB: 2M5G) and 1.4 Å (FimAa, PDB: 2JTY) for residues 20–158 (excluding the donor strand), compared with an RMSD of 2.0 Å for FimA in the crystal structure of the FimA-FimC complex (PDB: 4DWH), suggesting that the solution structures of uncomplexed FimA are more representative of the structure of FimA in the context of the fully assembled pilus and that FimC imposes a slight conformational change on FimA (Figures 2D and 2E). The main difference lies in a region of α-helix (residues 62–68) present in the FimA-FimC complex, which is absent and forms a loop in the structure of FimA inside the pilus (Figure 2E). In addition, there is a short 3_{10} helix (residues 25–28) present in FimA inside the pilus, which is absent in the FimA-FimC complex. The two NMR structures are very similar to FimA inside the pilus across residues 20–158, with perhaps the only difference being an additional short helix spanning residues 79–83 in FimAa (PDB: 2JTY), which is a loop in both the self-complemented FimA (PDB: 2M5G) and FimA inside the pilus (Figure 2D).

Comparison of the Type 1 and P Pilus Rods

Comparing and contrasting the near-atomic resolution models of the type 1 and P pili provides a complete picture of the general architecture of the archetypal chaperone-usher pili from UPEC. The pilins are arranged into similar right-handed superhelical quaternary assemblies, which differ in their helical rise and the number of subunits required to complete one turn (Figures 3A

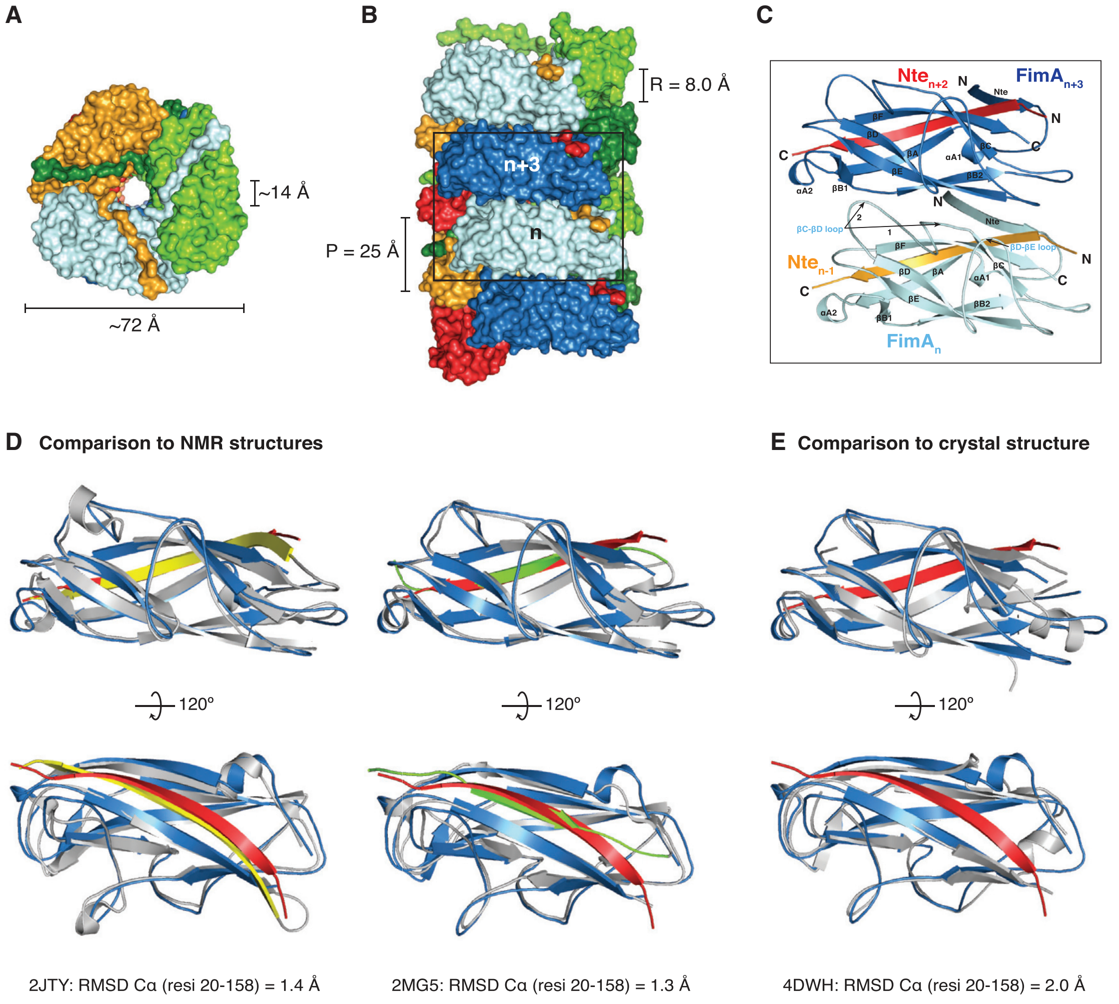

Figure 2. Overall Architecture of Type 1 Pili and Comparison of the FimA Pilin Structure

(A) Top view of the type 1 pilus rod model in surface representation. The last Nte of the light-green molecule has been removed for clarity. The dimensions of the overall and lumen diameters are indicated.

(B) Side view of the type 1 pilus rod model. There are three faces of the pilus rod structure, colored dark and light blue (front), dark and light green, and red and orange. A "stacking" interface is created between every n and n+3 subunit (boxed and labeled). The rise (R) and pitch (P) are indicated. For illustrative purposes, a model containing 12 molecules of FimA was created in PyMOL.

(C) Zoomed-in view of the stacking interface boxed in (B). The two FimA subunits are colored in light and dark blue and are shown in ribbon representation. Donor strands are colored orange and red, and key secondary structure elements are labeled. Residue-specific interaction details for this interface are shown in Figure S3.

(D) Superpositions of the FimA pilin from the fully assembled pilus structure (dark blue; Nte in red) and FimA structures determined by NMR spectroscopy (light gray). Left panel: comparison with FimA where the hydrophobic groove is complemented by a copy of the Nte fused to the C terminus (yellow) (PDB: 2JTY; Puorger et al., 2011). Right panel: comparison with FimAa where the hydrophobic groove is self-complemented by FimA's own Nte (green) (PDB: 2M5G; Walczak et al., 2014).

(E) Superposition of the FimA pilin from the fully assembled pilus structure (dark blue; Nte in red) and a crystal structure of FimA (light gray; Nte not shown) (PDB: 4DWH; Crespo et al., 2012). Two views rotated 120° with respect to each other are shown for each superposition. All proteins are shown in ribbon representation and the RMSD values for the alignment of Cα atoms for residues 2–158 are indicated.

Please refer to Figure S1 for the general architecture of UPEC chaperone-usher pili.

and 3B). The P and type 1 pilus structures have a similar pitch but contain 3.28 and 3.13 subunits per turn, respectively, making the P pilus slightly more tightly wound. This is also reflected in the slightly smaller helical rise per subunit of the P pilus structure (7.7 Å) compared with the type 1 pilus (8.0 Å) (Figures 3A and 3B). Both structures have an ascending path of Ntes that have their N-terminal ends facing the pilus exterior and their C-terminal ends lining the pilus lumen. The Nte of PapA is longer by one residue and its N-terminal portion (A1–P5), the so-called staple, creates a sharp ~90° angle with the remainder of the Nte (Hospenthal et al., 2016) (Figure 3C). By contrast, the N-terminal portion of the FimA Nte lies flat against the remainder of the FimA pilin fold. This difference is clear when the electron density of both the FimA and PapA Nte is compared, which allows the

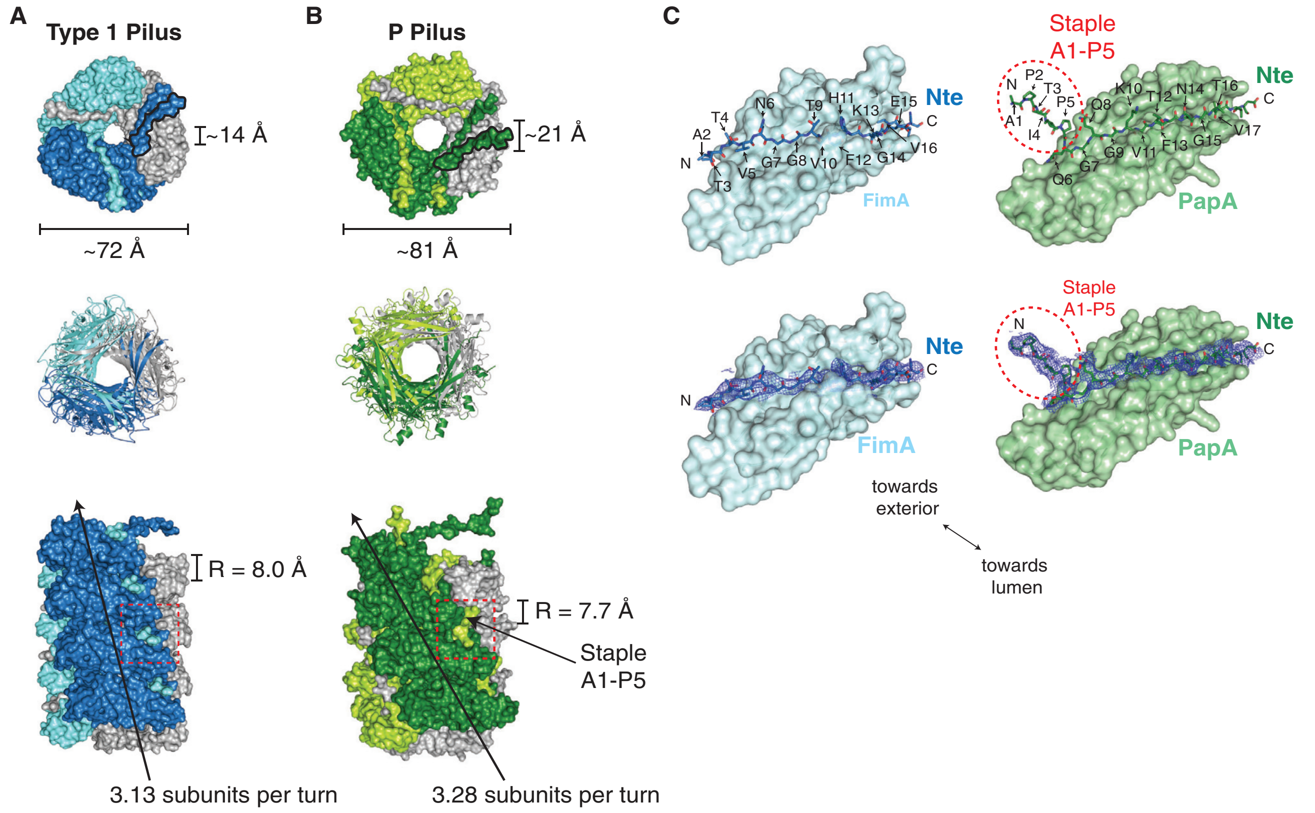

Figure 3. Comparison of the Type 1 and P Pilus Rod Structures

(A and B) Top and side views of (A) the type 1 pilus rod (colored dark blue, light blue, and gray) and (B) the P pilus rod (colored dark green, light green, and gray). The top view is shown in both surface and cartoon representation, whereas the side view is shown in surface representation. The last Nte of the top subunit in the surface representation of the top view, which does not undergo DSE with another subunit here, is outlined in black to distinguish it from the Nte of the same color emanating from the subunit below (n−3). Indicated are the dimensions of the outer and lumen diameters (top view), and the helical parameters of rise (R) and the number of subunits per turn (side view). A black arrow indicates the degree of twist in the pilus by tracing up the front face of the pilus structure. The N-terminal end of the Nte is visible between subunits (dashed red box) and is shorter and oriented differently in the type 1 pilus compared with the previously described "staple" region (residues 1–5) in the P pilus (Hospenthal et al., 2016). The model of the type 1 pilus begins at residue A2. For illustrative purposes, a model containing 12 molecules of FimA was created in PyMOL.

(C) A comparison between the Nte peptides complementing the pilin's hydrophobic groove in the type 1 pilus (left) and P pilus (right). The pilin subunits are shown in surface representation (FimA, light blue; PapA, light green) and the Nte peptide is shown in stick representation (FimA, dark blue; PapA, dark green). The Ntes of the pilin subunits shown in surface representation have been removed for clarity. The bottom panels show the quality of the electron density surrounding the Nte peptides, illustrating the differences at the N terminus. The PapA Nte makes a sharp turn and forms the "staple" region (red dashed ellipse), whereas the FimA Nte lies flat against the FimA subunit. Residues are labeled and an arrow indicates the overall orientation of the pilins.

Please refer to Figure S1 for the general architecture of UPEC chaperone-usher pili.

main chain to be traced unambiguously in both structures (Figure 3C).

Comparison of Cryo-EM and ssNMR/STEM Structures of the Type 1 Pilus Rod

A different model of the type 1 pilus rod structure has been reported recently using a hybrid approach of solid-state NMR spectroscopy (ssNMR) and scanning transmission electron microscopy (STEM) (Habenstein et al., 2015) (Figure 4). This model has a helical arrangement of 3.46 FimA subunits per turn with a rise of 7.2 Å (Figure 4B), which agrees with a previous low-resolution EM study (Hahn et al., 2002). Such a discrepancy leads to significant offsets observed in the stacking interface between the n and n+3 subunits (Figure 4C).

The type 1 pilus preparations used for our present cryo-EM structure and the previous ssNMR/STEM structure were obtained according to different protocols. While native pili, assembled by the usher FimD *in vivo*, were used for our cryo-EM structure, the pili used for the ssNMR/STEM structure had been produced in an *in vitro* assembly reaction in which FimA-FimC complexes reacted spontaneously into pilus rods and free FimC (Habenstein et al., 2015). To test whether differences in the cryo-EM and ssNMR/STEM structures could be a consequence of differences in the quaternary structures between pili assembled *in vivo* and *in vitro*, we produced type 1 pili *in vitro* through spontaneous assembly of FimA monomers (see STAR Methods for the detailed protocol). As kinetic stability against unfolding is a very sensitive parameter for quantifying the ratios between spectroscopically indistinguishable protein conformations differing in stability (Schmid, 1983), we hypothesized that pili assembled *in vivo* and *in vitro* should differ in their kinetic stability against dissociation and unfolding by denaturants, even if

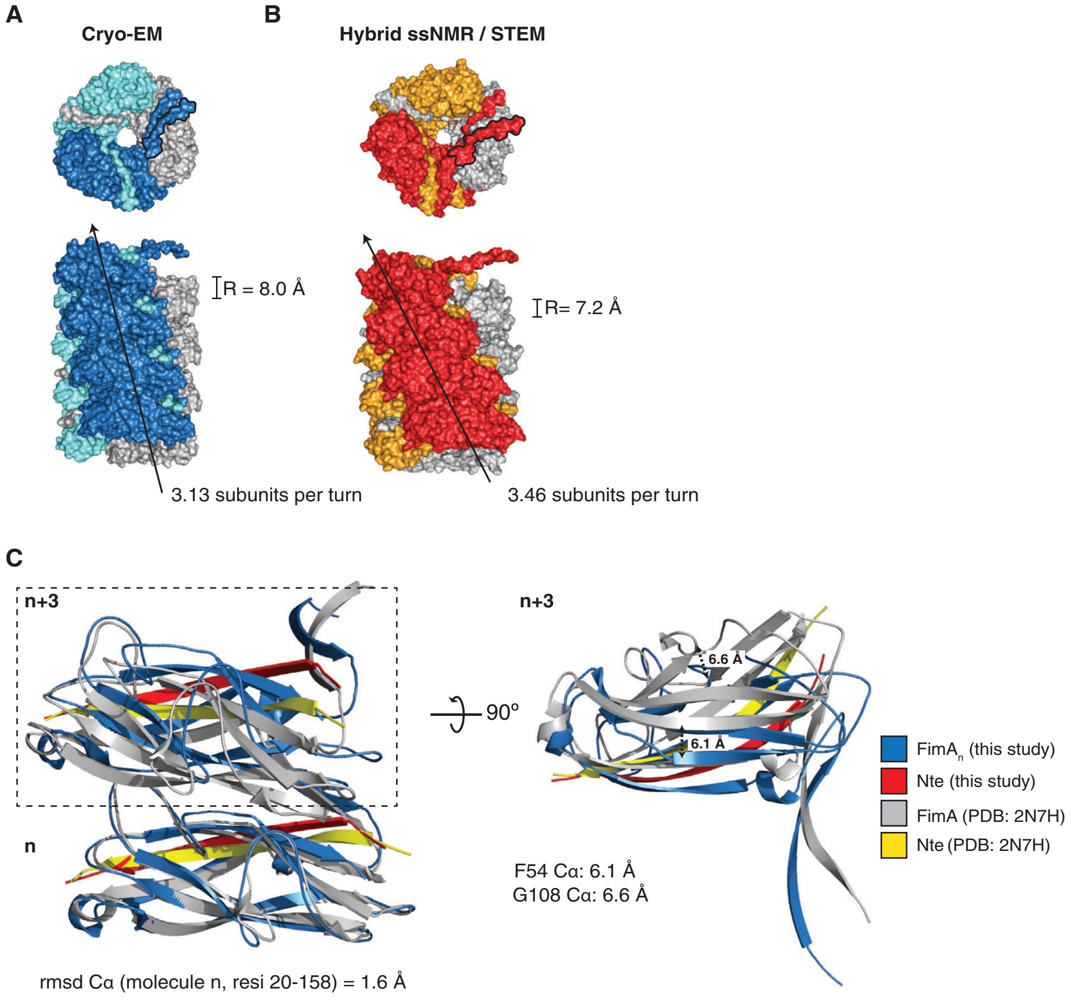

Figure 4. Comparison of the Type 1 Pilus Rod Structure Determined by Cryo-EM and a Hybrid ssNMR/STEM Approach

(A) Top view (upper panel) and side view (lower panel) of the cryo-EM-derived model of the type 1 pilus rod shown in surface representation. The pilus subunits are colored as in Figure 3A. A black arrow indicates the degree of twist in the pilus by tracing up the front face of the pilus structure. The helical parameter of rise (R) and the number of subunits per turn are indicated. For illustrative purposes, a model containing 12 molecules of FimA was created in PyMOL.

(B) The hybrid model derived from ssNMR and STEM data (colored red, orange, and gray) shown in surface representation and labeled as in (A). The number of subunits per turn was calculated by dividing the pitch (24.9 Å) by the rise (7.2 Å) (Habenstein et al., 2015). The last Nte of the top subunit in the surface representation of the top view, which does not undergo DSE with another subunit, is outlined in black to distinguish it from the Nte of the same color emanating from the subunit below (n−3).

(C) Left panel: superposition of the two subunits participating in the pilus' main stacking interface (n and n+3) of the cryo-EM-derived model (blue, Nte in red) and the ssNMR/STEM-derived model (gray, Nte in yellow). The n subunit was aligned and the RMSD value for the alignment of Cα atoms for residues 2–158 is indicated below. Right panel: 90° rotation of the n+3 subunit showing the offset of key β strands and loops in the stacking interface as a result of the differences in twist and rise. The distances between equivalent Cα atoms (F54 and G108) are indicated.

Please refer to Figure S1 for the general architecture of UPEC chaperone-usher pili.

they only had slightly different quaternary structures. Figure 5 shows the guanidinium chloride (GdmCl)-dependent dissociation/unfolding kinetics at pH 2.1 for both pilus preparations, recorded via the decrease in the far-UV circular dichroism (CD) signal at 230 nm. The results demonstrated that native pili formed *in vivo* were indeed significantly more stable against dissociation and unfolding than pili assembled *in vitro* and also differed in the denaturant sensitivity of the rate constant of dissociation/unfolding (Figure 5). Specifically, the extrapolated unfolding rate constants of pili assembled *in vivo* proved to be 3–4 orders of magnitude smaller compared with the unfolding rates of pili assembled *in vitro* recorded in the range of 5.8–6.4 M GdmCl (Figure 5). In addition, both pilus preparations proved to be homogeneous, as all unfolding traces could be fitted with a single exponential function (see STAR Methods; Figures 5 and S4). We conclude that type 1 pili can adopt different quaternary structures and that the mechanism and the conditions of the assembly reaction likely define the specific quaternary structure of the pilus rod. In addition, formation of a specific pilus conformer appears to be irreversible. The differences between the cryo-EM and the ssNMR/STEM structures of type 1 pili might thus indeed result from different quaternary structures.

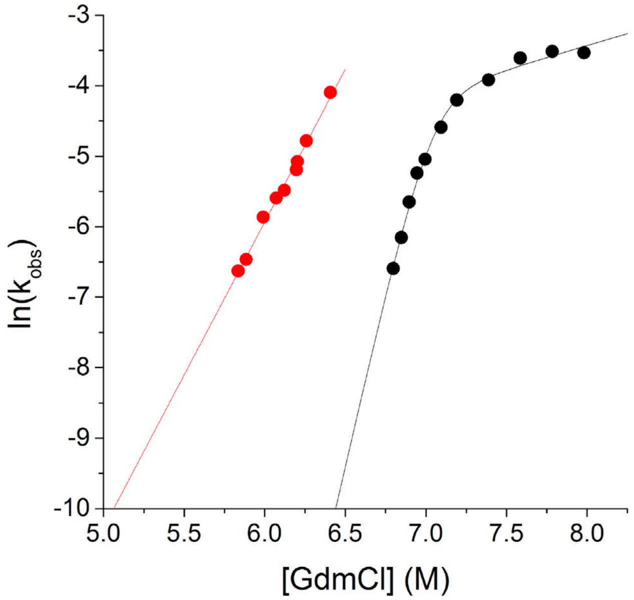

Figure 5. Kinetic Stability Against Dissociation/Unfolding by GdmCl at pH 2.1 and 25°C of Type 1 Pili Assembled *In Vivo* and *In Vitro*

The kinetics of dissociation/unfolding of type 1 pili assembled *in vivo* (black symbols) or *in vitro* (red symbols) at pH 2.1 and different GdmCl concentrations were followed via the decrease in the far-UV CD signal of FimA upon unfolding. All kinetic traces were fully consistent with a single first-order reaction, showing that the pilus preparations were homogeneous and did not consist of mixtures of pili with different stability against unfolding/dissociation. The logarithms of the rate constants of dissociation/unfolding (k_{obs}) were plotted against GdmCl concentration. The results show that pili assembled *in vivo* are clearly more stable than those assembled *in vitro*. The differences in the slopes of the GdmCl dependence of k_{obs} indicate a higher solvent accessibility of the transition state of dissociation/unfolding for the pili assembled *in vivo*. Please refer to Figure S1 for the general architecture of UPEC chaperone-usher pili and Figure S4 for further details about type 1 pilus rod unfolding/dissociation.

DISCUSSION

This near-atomic resolution model of the type 1 pilus rod allows us to fully appreciate the similarities and differences of the two archetypal chaperone-usher pili of UPEC. FimA and PapA are related proteins with 31.7% sequence identity (mature proteins), sharing the same C-terminally truncated Ig-like pilin fold, and are assembled by related members of the chaperone-usher pathway. Not surprisingly, the quaternary structure formed by FimA or PapA, the pilus rod, also shares several similarities. Both the type 1 and P pilus rods form right-handed superhelical arrangements of similar overall dimensions (Figures 3A and 3B). The P pilus rod is formed of 3.28 subunits per turn with an axial rise of 7.7 Å, whereas the type 1 pilus rod is formed of 3.13 subunits per turn with a rise of 8.0 Å. These differences mean that the P pilus adopts a more "twisted" or tightly wound conformation (Figure 3B). Furthermore, the type 1 and P pilus rod structures differ in their Nte regions. The five N-terminal residues (A1–P5) of PapA form the "staple" region, which forms a ~90° angle with respect to the remainder of the Nte (Figure 3C). This difference in the conformation (and length) of the Nte allows the staple residues of PapA to reach and make contact with the n+5 subunit, thereby creating an interaction network where each subunit contacts 10 others (two more than in the type 1 pilus) (Hospenthal et al., 2016).

However, as demonstrated for the P pilus, the most important interface is the main stacking interface formed between every n and n+3 subunit (Figures 2C and S3A). This interface is responsible for maintaining the quaternary structural integrity of the pilus and also governs the biomechanical properties of reversible uncoiling in response to shear forces such as those experienced in the urinary tract. Chaperone-usher pili have been the subjects of several studies utilizing force spectroscopy techniques such as optical tweezers or AFM (Andersson et al., 2008, 2007, 2006c, 2006b, 2006a; Fällman et al., 2005; Forero et al., 2006; Jass et al., 2004; Lugmaier et al., 2007; Miller et al., 2006; Zakrisson et al., 2013, 2012). A mathematical model of the force versus elongation behavior of P pili was developed, identifying three elongation regions (Jass et al., 2004). Region I is characterized by a linear force versus elongation response and is thought to reflect the elastic stretching of the quaternary rod structure (although not yet breaking it). Region II results in elongation under constant force and represents the sequential opening of the stack-to-stack interactions resulting in rod unwinding. Finally, Region III shows an "s-shaped" force versus elongation response and represents the overstretching of the now linearized rod, still held together by intermolecular DSE interactions. Both regions I and II depend on the interface created by the n and n+3 subunits. The unwinding of the rod in region II occurs either under steady-state or dynamic conditions depending on the elongation speed applied (Andersson et al., 2008, 2007). Measurements performed under steady-state conditions revealed that type 1 and P pili unwind at comparable unfolding forces (28 ± 2 and 30 ± 2 pN, respectively) (Andersson et al., 2007). However, measurements performed under dynamic conditions (dynamic force spectroscopy) can address values of physical entities that steady-state measurements cannot address, such as the bond-opening rate and bond lengths ("bond" here refers to the stack-to-stack interactions in the quaternary rod structure) (Andersson et al., 2008, 2007). Such measurements have suggested that a higher force is required to unwind type 1 pili compared with P pili at these fast elongation rates. In turn, this implies that the stacking interface is stronger in type 1 pili (Andersson et al., 2008, 2007). These findings are indeed supported by our cryo-EM structure of the type 1 pilus rod, which has a larger buried surface area (1,616.2 $Å^2$) in the n and n+3 (stacking) interface than the P pilus (1,453.0 $Å^2$) (Hospenthal et al., 2016) (Figure S3B). This larger buried surface area may explain the lower thermal bond-opening rate observed for type 1 pilus rods.

Interestingly, two different recoiling forces have been observed for type 1 pili, suggesting that type 1 pili can rearrange into two distinct quaternary structures after having been extended and linearized (Andersson et al., 2008, 2007). This raises the intriguing question of whether the two different structures of type 1 pili, the cryo-EM structure (this study) and the hybrid ssNMR/STEM model (Habenstein et al., 2015), and/or the two different conformers detected by GdmCl-dependent unfolding kinetics (Figure 5), may represent the two different states observed in these force spectroscopy experiments.

Chaperone-usher pili of Gram-negative pathogens are biopolymers with remarkable biomechanical properties. The

availability of near-atomic resolution models of both the type 1 and P pilus rods provides an unprecedented opportunity to understand which factors govern their differences when it comes to reversible rod uncoiling. Although these structures appear superficially similar, subtle differences in key interfaces and in their helical parameters could influence how these structures behave in response to shear forces during host infection. The work described here, together with that of the P pilus (Hospenthal et al., 2016), now provides the basis on which accurate molecular dynamics simulations can be implemented that will probe the energetic, structural, and molecular basis of pilus uncoiling and recoiling.

STAR★METHODS

Detailed methods are provided in the online version of this paper and include the following:

- KEY RESOURCES TABLE
- CONTACT FOR REAGENT AND RESOURCE SHARING
- EXPERIMENTAL MODEL AND SUBJECT DETAILS
- METHOD DETAILS
  - Molecular Biology
  - Pilus Expression and Purification for Cryo-EM
  - Production and Purification of FimA Monomers
  - *In Vitro* Assembly of Type 1 Pilus Rods
  - *In Vivo* Pilus Assembly for Stability Studies
  - Dissociation and Unfolding Kinetics
  - Cryo-EM Sample Preparation and Data Collection
  - Cryo-EM Image Processing and Reconstruction
  - Model Building, Refinement, Structure Analysis
- QUANTIFICATION AND STATISTICAL ANALYSIS
- DATA AND SOFTWARE AVAILABILITY

SUPPLEMENTAL INFORMATION

Supplemental Information includes four figures and one table and can be found with this article online at https://doi.org/10.1016/j.str.2017.10.004.

AUTHOR CONTRIBUTIONS

M.K.H. purified pili, prepared cryo-EM grids, collected data, performed image processing and reconstruction, built models, made figures, and wrote the paper. T.R.D.C. and A.R. helped with screening conditions and vitrifying of cryo-EM grids. T.R.D.C. supervised EM data collection. J.L. cloned the pSH5 plasmid. C.G. and D.Z. purified pili and evaluated dissociation/unfolding kinetics. D.Z. performed *in vitro* assembly of FimA. R.G. and G.W. supervised the work and wrote the paper.

ACKNOWLEDGMENTS

This work was funded by MRC grant 018434 to G.W. and by the Swiss National Science Foundation grant 31003A_156304 to R.G. We wish to thank Dr. Natasha Lukoyanova for assistance with EM data collection and Shaoda He for advice on image processing in RELION-2.0. We also wish to thank Dr. Aravindan Ilangovan and Germán Sgro for advice on model building and refinement.

Received: July 26, 2017
Revised: October 5, 2017
Accepted: October 23, 2017
Published: November 9, 2017

REFERENCES

Adams, P.D., Afonine, P.V., Bunkoczi, G., Chen, V.B., Davis, I.W., Echols, N., Headd, J.J., Hung, L.W., Kapral, G.J., Grosse-Kunstleve, R.W., et al. (2010). PHENIX: a comprehensive Python-based system for macromolecular structure solution. Acta Crystallogr. D Biol. Crystallogr. *66*, 213–221.

Andersson, M., Axner, O., Almqvist, F., Uhlin, B.E., and Fällman, E. (2008). Physical properties of biopolymers assessed by optical tweezers: analysis of folding and refolding of bacterial pili. ChemPhysChem *9*, 221–235.

Andersson, M., Fällman, E., Uhlin, B.E., and Axner, O. (2006a). A sticky chain model of the elongation and unfolding of *Escherichia coli* P pili under stress. Biophys. J. *90*, 1521–1534.

Andersson, M., Fällman, E., Uhlin, B.E., and Axner, O. (2006b). Dynamic force spectroscopy of *E. coli* P pili. Biophys. J. *91*, 2717–2725.

Andersson, M., Fällman, E., Uhlin, B.E., and Axner, O. (2006c). Force measuring optical tweezers system for long time measurements of P pili stability. In Proceedings of SPIE-Imaging, Manipulation, and Analysis of Biomolecules, Cells, and Tissue IV, *6088*, D.L. Farkas, D.V. Nicolau, and R.C. Leif, eds. (SPIE Publications). https://doi.org/10.1117/12.642266.

Andersson, M., Uhlin, B.E., and Fällman, E. (2007). The biomechanical properties of *E. coli* pili for urinary tract attachment reflect the host environment. Biophys. J. *93*, 3008–3014.

Bachmann, A., and Kiefhaber, T. (2001). Apparent two-state tendamistat folding is a sequential process along a defined route. J. Mol. Biol. *306*, 375–386.

Bachmann, B.J. (1990). Linkage map of *Escherichia coli* K-12, edition 8. Microbiol. Rev. *54*, 130–197.

Barnhart, M.M., Pinkner, J.S., Soto, G.E., Sauer, F.G., Langermann, S., Waksman, G., Frieden, C., and Hultgren, S.J. (2000). PapD-like chaperones provide the missing information for folding of pilin proteins. Proc. Natl. Acad. Sci. USA *97*, 7709–7714.

Chen, V.B., Arendall, W.B., Headd, J.J., Keedy, D.A., Immormino, R.M., Kapral, G.J., Murray, L.W., Richardson, J.S., and Richardson, D.C. (2010). MolProbity: all-atom structure validation for macromolecular crystallography. Acta Crystallogr. D Biol. Crystallogr. *66*, 12–21.

Choudhury, D., Thompson, A., Stojanoff, V., Langermann, S., Pinkner, J., Hultgren, S.J., and Knight, S.D. (1999). X-ray structure of the FimC-FimH chaperone-adhesin complex from uropathogenic *Escherichia coli*. Science *285*, 1061–1066.

Colón, W. (1999). Analysis of protein structure by solution optical spectroscopy. Methods Enzymol. *309*, 605–632.

Crespo, M.D., Puorger, C., Schärer, M.A., Eidam, O., Grütter, M.G., Capitani, G., and Glockshuber, R. (2012). Quality control of disulfide bond formation in pilus subunits by the chaperone FimC. Nat. Chem. Biol. *8*, 707–713.

Dodson, K.W., Pinkner, J.S., Rose, T., Magnusson, G., Hultgren, S.J., and Waksman, G. (2001). Structural basis of the interaction of the pyelonephritic *E. coli* adhesin to its human kidney receptor. Cell *105*, 733–743.

Ellman, G.L. (1959). Tissue sulfhydryl groups. Arch. Biochem. Biophys. *82*, 70–77.

Emsley, P., Lohkamp, B., Scott, W.G., and Cowtan, K. (2010). Features and development of Coot. Acta Crystallogr. D Biol. Crystallogr. *D66*, 486–501.

Fällman, E., Schedin, S., Jass, J., Uhlin, B.E., and Axner, O. (2005). The unfolding of the P pili quaternary structure by stretching is reversible, not plastic. EMBO Rep. *6*, 52–56.

Flores-Mireles, A.L., Walker, J.N., Caparon, M., and Hultgren, S.J. (2015). Urinary tract infections: epidemiology, mechanisms of infection and treatment options. Nat. Rev. Microbiol. *13*, 269–284.

Forero, M., Yakovenko, O., Sokurenko, E.V., Thomas, W.E., and Vogel, V. (2006). Uncoiling mechanics of *Escherichia coli* type I fimbriae are optimized for catch bonds. PLoS Biol. *4*, e298–e299.

Geibel, S., Procko, E., Hultgren, S.J., Baker, D., and Waksman, G. (2013). Structural and energetic basis of folded-protein transport by the FimD usher. Nature *496*, 243–246.

Giese, C., Zosel, F., Puorger, C., and Glockshuber, R. (2012). The most stable protein-ligand complex: applications for one-step affinity purification and identification of protein assemblies. Angew. Chem. Int. Ed. *51*, 4474–4478.

Habenstein, B., Loquet, A., Hwang, S., Giller, K., Vasa, S.K., Becker, S., Habeck, M., and Lange, A. (2015). Hybrid structure of the type 1 pilus of uropathogenic *Escherichia coli*. Angew. Chem. Int. Ed. *127*, 11857–11861.

Hahn, E., Wild, P., Hermanns, U., Sebbel, P., Glockshuber, R., Häner, M., Taschner, N., Burkhard, P., Aebi, U., and Müller, S.A. (2002). Exploring the 3D molecular architecture of *Escherichia coli* type 1 pili. J. Mol. Biol. *323*, 845–857.

Hannan, T.J., Totsika, M., Mansfield, K.J., Moore, K.H., Schembri, M.A., and Hultgren, S.J. (2012). Host-pathogen checkpoints and population bottlenecks in persistent and intracellular uropathogenic *Escherichia coli* bladder infection. FEMS Microbiol. Rev. *36*, 616–648.

He, S., and Scheres, S.H.W. (2017). Helical reconstruction in RELION. J. Struct. Biol. *198*, 163–176.

Holm, L., and Laakso, L.M. (2016). Dali server update. Nucleic Acids Res. *44*, W351–W355.

Horton, R.M., Hunt, H.D., Ho, S.N., Pullen, J.K., and Pease, L.R. (1989). Engineering hybrid genes without the use of restriction enzymes: gene splicing by overlap extension. Gene *77*, 61–68.

Hospenthal, M.K., Costa, T.R.D., and Waksman, G. (2017). A comprehensive guide to pilus biogenesis in Gram-negative bacteria. Nat. Rev. Microbiol. *15*, 365–379.

Hospenthal, M.K., Redzej, A., Dodson, K., Ukleja, M., Frenz, B., Rodrigues, C., Hultgren, S.J., DiMaio, F., Egelman, E.H., and Waksman, G. (2016). Structure of a chaperone-usher pilus reveals the molecular basis of rod uncoiling. Cell *164*, 269–278.

Jass, J., Schedin, S., Fällman, E., Ohlsson, J., Nilsson, U.J., Uhlin, B.E., and Axner, O. (2004). Physical properties of *Escherichia coli* P pili measured by optical tweezers. Biophys. J. *87*, 4271–4283.

Joosten, R.P., te Beek, T.A., Krieger, E., Hekkelman, M.L., Hooft, R.W., Schneider, R., Sander, C., and Vriend, G. (2010). A series of PDB related databases for everyday needs. Nucleic Acids Res. *39*, D411–D419.

Kabsch, W., and Sander, C. (1983). Dictionary of protein secondary structure: pattern recognition of hydrogen-bonded and geometrical features. Biopolymers *22*, 2577–2637.

Link, A.J., Phillips, D., and Church, G.M. (1997). Methods for generating precise deletions and insertions in the genome of wild-type *Escherichia coli*: applications to open reading frame characterization. J. Bacteriol. *179*, 6228–6237.

Lugmaier, R.A., Schedin, S., Kühner, F., and Benoit, M. (2007). Dynamic restacking of *Escherichia coli* P-pili. Eur. Biophys. J. *37*, 111–120.

McLellan, L.K., and Hunstad, D.A. (2016). Urinary tract infection: pathogenesis and outlook. Trends Mol. Med. *22*, 946–957.

Miller, E., Garcia, T., Hultgren, S., and Oberhauser, A.F. (2006). The mechanical properties of *E. coli* type 1 pili measured by atomic force microscopy techniques. Biophys. J. *91*, 3848–3856.

Mulvey, M.A., Lopez-Boado, Y.S., Wilson, C.L., Roth, R., Parks, W.C., Heuser, J., and Hultgren, S.J. (1998). Induction and evasion of host defenses by type 1-piliated uropathogenic *Escherichia coli*. Science *282*, 1494–1497.

Nishiyama, M., Ishikawa, T., Rechsteiner, H., and Glockshuber, R. (2008). Reconstitution of pilus assembly reveals a bacterial outer membrane catalyst. Science *320*, 376–379.

Nozaki, Y. (1972). The preparation of guanidine hydrochloride. Methods Enzymol. *26*, 43–50.

Orndorff, P.E., and Falkow, S. (1984). Identification and characterization of a gene product that regulates type 1 piliation in *Escherichia coli*. J. Bacteriol. *160*, 61–66.

Pettersen, E.F., Goddard, T.D., Huang, C.C., Couch, G.S., Greenblatt, D.M., Meng, E.C., and Ferrin, T.E. (2004). UCSF Chimera—a visualization system for exploratory research and analysis. J. Comput. Chem. *25*, 1605–1612.

Phan, G., Remaut, H., Wang, T., Allen, W.J., Pirker, K.F., Lebedev, A., Henderson, N.S., Geibel, S., Volkan, E., Yan, J., et al. (2011). Crystal structure of the FimD usher bound to its cognate FimC-FimH substrate. Nature *474*, 49–53.

Puorger, C., Vetsch, M., Wider, G., and Glockshuber, R. (2011). Structure, folding and stability of FimA, the main structural subunit of type 1 pili from uropathogenic *Escherichia coli* strains. J. Mol. Biol. *412*, 520–535.

Remaut, H., Tang, C., Henderson, N.S., Pinkner, J.S., Wang, T., Hultgren, S.J., Thanassi, D.G., Waksman, G., and Li, H. (2008). Fiber formation across the bacterial outer membrane by the chaperone/usher pathway. Cell *133*, 640–652.

Roberts, J.A., Marklund, B.I., Ilver, D., Haslam, D., Kaack, M.B., Baskin, G., Louis, M., Möllby, R., Winberg, J., and Normark, S. (1994). The Gal(alpha 1-4)Gal-specific tip adhesin of *Escherichia coli* P-fimbriae is needed for pyelonephritis to occur in the normal urinary tract. Proc. Natl. Acad. Sci. USA *91*, 11889–11893.

Rosenthal, P.B., and Henderson, R. (2003). Optimal determination of particle orientation, absolute hand, and contrast loss in single-particle electron cryomicroscopy. J. Mol. Biol. *333*, 721–745.

Sauer, F.G., Fütterer, K., Pinkner, J.S., Dodson, K.W., Hultgren, S.J., and Waksman, G. (1999). Structural basis of chaperone function and pilus biogenesis. Science *285*, 1058–1061.

Sauer, F.G., Pinkner, J.S., Waksman, G., and Hultgren, S.J. (2002). Chaperone priming of pilus subunits facilitates a topological transition that drives fiber formation. Cell *111*, 543–551.

Sauer, F.G., Remaut, H., Hultgren, S.J., and Waksman, G. (2004). Fiber assembly by the chaperone-usher pathway. Biochim. Biophys. Acta *1694*, 259–267.

Scheres, S.H. (2012). RELION: implementation of a Bayesian approach to cryo-EM structure determination. J. Struct. Biol. *180*, 519–530.

Schmid, F.X. (1983). Mechanism of folding of ribonuclease A. Slow refolding is a sequential reaction via structural intermediates. Biochemistry *22*, 4690–4696.

Spaulding, C., and Hultgren, S. (2016). Adhesive pili in UTI pathogenesis and drug development. Pathogens *5*, 30.

Stathopoulos, C., Hultgren, S.J., Thanassi, D.G., Hendrixson, D.R., St Geme, J.W., III, and Curtiss, R., III (2000). Secretion of virulence determinants by the general secretory pathway in Gram-negative pathogens: an evolving story. Microbes Infect. *2*, 1061–1072.

Studier, F.W., Rosenberg, A.H., Dunn, J.J., and Dubendorff, J.W. (1990). Use of T7 RNA polymerase to direct expression of cloned genes. Methods Enzymol. *185*, 60–89.

Thanassi, D.G., Bliska, J.B., and Christie, P.J. (2012). Surface organelles assembled by secretion systems of Gram-negative bacteria: diversity in structure and function. FEMS Microbiol. Rev. *36*, 1046–1082.

Thanassi, D.G., Saulino, E.T., and Hultgren, S.J. (1998). The chaperone/usher pathway: a major terminal branch of the general secretory pathway. Curr. Opin. Microbiol. *1*, 223–231.

Vagin, A.A., Steiner, R.A., Lebedev, A.A., Potterton, L., McNicholas, S., Long, F., and Murshudov, G.N. (2004). REFMAC5 dictionary: organization of prior chemical knowledge and guidelines for its use. Acta Crystallogr. D Biol. Crystallogr. *60*, 2184–2195.

Vangone, A., Spinelli, R., Scarano, V., Cavallo, L., and Oliva, R. (2011). COCOMAPS: a web application to analyze and visualize contacts at the interface of biomolecular complexes. Bioinformatics *27*, 2915–2916.

Vetsch, M., Puorger, C., Spirig, T., Grauschopf, U., Weber-Ban, E.U., and Glockshuber, R. (2004). Pilus chaperones represent a new type of protein-folding catalyst. Nature *431*, 329–333.

Waksman, G. (2017). Structural and molecular biology of a protein-polymerising nanomachine for pilus biogenesis. J. Mol. Biol. *429*, 2654–2666.

Waksman, G., and Hultgren, S.J. (2009). Structural biology of the chaperone-usher pathway of pilus biogenesis. Nat. Rev. Microbiol. *7*, 765–774.

Walczak, M.J., Puorger, C., Glockshuber, R., and Wider, G. (2014). Intramolecular donor strand complementation in the *E. coli* Type 1 pilus

subunit FimA explains the existence of FimA monomers as off-pathway products of pilus assembly that inhibit host cell apoptosis. J. Mol. Biol. *426*, 542–549.

Zakrisson, J., Wiklund, K., Axner, O., and Andersson, M. (2013). The shaft of the type 1 fimbriae regulates an external force to match the FimH catch bond. Biophys. J. *104*, 2137–2148.

Zakrisson, J., Wiklund, K., Axner, O., and Andersson, M. (2012). Helix-like biopolymers can act as dampers of force for bacteria in flows. Eur. Biophys. J. *41*, 551–560.

Zavialov, A.V., Berglund, J., Pudney, A.F., Fooks, L.J., Ibrahim, T.M., MacIntyre, S., and Knight, S.D. (2003). Structure and biogenesis of the capsular F1 antigen from *Yersinia pestis*: preserved folding energy drives fiber formation. Cell *113*, 587–596.

Zhang, K. (2016). Gctf: real-time CTF determination and correction. J. Struct. Biol. *193*, 1–12.

Zheng, S.Q., Palovcak, E., Armache, J.-P., Verba, K.A., Cheng, Y., and Agard, D.A. (2017). MotionCor2: anisotropic correction of beam-induced motion for improved cryo-electron microscopy. Nat. Methods *14*, 331–332.

STAR★METHODS

KEY RESOURCES TABLE

REAGENT or RESOURCE	SOURCE	IDENTIFIER
Bacterial and Virus Strains		
Escherichia coli HB101	Promega	Cat#L2011
Escherichia coli W3110Δ*fimA*	This study	N/A
Escherichia coli BL21 (DE3)	New England Biolabs	Cat#C2527
Deposited Data		
Type 1 Pilus Rod cryo-EM map	This study	EMD-3809
Type 1 Pilus Rod cryo-EM structure (model)	This study	PDB ID: 5OH0
Oligonucleotides		
Primer p1: 5'-ACC TCC GAA CGT CATATG AAA ATT AAA ACT TGG CAA TCG-3'	Microsynth	N/A
Primer p2: 5'-CGT TAT TTT TAT CGC ACA AGGG-3'	Microsynth	N/A
Primer p3: 5'-CCC TTG TGC GAT AAA AAT AAC GGT GAG TAA AAA AAC GTC AAT GTA AGG-3'	Microsynth	N/A
Primer p4: 5'-ACC TCC GAA CACTAGT TAT TCC ATT ACG CCC GTC-3'	Microsynth	N/A
Primer p5: 5'-CTC CGA ACG TGCATGC GCG CAA CGC AAT TAA TGT AAG-3'	Microsynth	N/A
Primer p6: 5'-ACC TAC TAA CACTAGT GGC TGC TAA CAA AGC CCG-3'	Microsynth	N/A
Recombinant DNA		
pSH2	Orndorff and Falkow, 1984	N/A
pSH5	This study	N/A
pCG1-AC	This study	N/A
pFimAwt_cyt	Puorger et al., 2011	N/A
Software and Algorithms		
MOTIONCOR2	Zheng et al., 2017	http://msg.ucsf.edu/em/software/motioncor2.html
GCTF	Zhang, 2016	http://www.mrc-lmb.cam.ac.uk/kzhang/
RELION-2.0	Scheres, 2012; He and Scheres, 2017	http://www2.mrc-lmb.cam.ac.uk/relion/index.php/Main_Page
Chimera	Pettersen et al., 2004	https://www.cgl.ucsf.edu/chimera/
Coot	Emsley et al., 2010	https://www2.mrc-lmb.cam.ac.uk/personal/pemsley/coot/
Phenix	Adams et al., 2010	https://www.phenix-online.org
Refmac5	Vagin et al., 2004	http://www.ccp4.ac.uk/html/refmac5.html
Molprobity	Chen et al., 2010	https://www.phenix-online.org
CoComaps	Vangone et al., 2011	https://www.molnac.unisa.it/BioTools/cocomaps/
DSSP server	Joosten et al., 2010; Kabsch and Sander, 1983	http://www.cmbi.ru.nl/dssp.html; http://swift.cmbi.ru.nl/gv/dssp/
wwPDB validation Service	wwPDB	https://validate-rcsb-1.wwpdb.org/
DALI server	Holm and Laakso, 2016	http://ekhidna.biocenter.helsinki.fi/dali_server/start
OriginPro 2017	Origin (OriginLab, Northampton, MA)	http://www.originlab.com/
Spectragryph 1.2.4	Dr. Friedrich Menges Software-Entwicklung	http://spectroscopy.ninja/

CONTACT FOR REAGENT AND RESOURCE SHARING

Further information and requests for resources and reagents should be directed to and will be fulfilled by the Lead Contact, Gabriel Waksman (g.waksman@mail.cryst.bbk.ac.uk or g.waksman@ucl.ac.uk)

EXPERIMENTAL MODEL AND SUBJECT DETAILS

HB101 and BL21 (DE3) *E. coli* cells were cultured in Luria-Bertani (LB) medium at 37°C, while the W3110Δ*fimA E. coli* strain was grown in 2YT medium at 37°C supplemented with anhydrotetracycline (12.5 ng/ml). The media was further supplemented with antibiotics after the transformation of a plasmid for subsequent protein expression (see below). All bacterial cultures were grown in a shaking incubator.

METHOD DETAILS

Molecular Biology

The pSH2 plasmid harbouring the *fim* operon from the UPEC strain J96 has been described previously (Orndorff and Falkow, 1984). An arabinose-inducible plasmid, pSH5, was created by subcloning the *FimA* to *FimH* gene cluster from pSH2 onto the pBADM-11 vector backbone. pSH5 (Amp^R) was transformed into HB101 *E. coli* cells (Promega) for cell-surface pilus production.

The W3110Δ*fimA* strain (unmarked deletion) was created by deleting bp 4-528 of the *fimA* gene from the chromosome of the E. *coli* K12 wild type strain W3110 (B. J. Bachmann, 1990) as described (Link et al., 1997).

Plasmid pCG1-AC for periplasmic co-expression of FimA and FimC under *tet* promoter control was constructed by ligation of three DNA fragments: Fragment 1 encoding FimA and FimC was obtained by PCR (template: genomic DNA of *E. coli* W3110) and overlap extension (Horton et al., 1989) using primers p1, p2, p3 and p4. Fragment 2 was obtained by PCR using pET-11a (Studier et al., 1990) as template and primers p5 and p6 Fragment 3, harboring the *tetA* promoter and the *tetR* gene, was obtained by digesting plasmid pDsF-TrpA (Giese et al., 2012) with NdeI and SphI. The final construct pCG1-AC contains the *tetA* promoter, the *tetR*, *bla*, *fimA* and *fimC* genes and a pBR322 origin of replication. The correct DNA sequence of pCG1-AC was confirmed by dideoxy DNA sequencing.

Pilus Expression and Purification for Cryo-EM

6 L cultures of HB101 *E. coli* cells, transformed with pSH5, were grown to optical density at 600 nm (OD_{600}) of 0.6-0.8 in LB medium supplemented with ampicillin (100 μg/mL) at 37°C in a shaking incubator. The expression of type 1 chaperone-usher pili was induced with 0.05% (w/v) arabinose and the culture was incubated further for 1 h at 37°C before harvesting the cells by centrifugation. Pili were sheared off the cell surface by gently stirring the cells for 2 h resuspended in 400 mL of buffered solution containing 30 mM sodium citrate dehydrate [pH 7.2], 300 mM NaCl, 1 mg/mL DNase and cOmplete™ mini EDTA-free protease inhibitor cocktail tablets (Roche, 1 tablet/200 mL). Subsequently, depiliated cells were removed by two rounds of centrifugation at 10'800 x *g* for 30 min (SLA-300 rotor; Sorvall) and pili were precipitated by incubating and gently stirring the supernatant in the presence of 5% (w/v) PEG 6000, 0.5 M NaCl for 30 min. The precipitate was pelleted by centrifugation at 18'000 x *g* for 30 min (SLA-3000; Sorvall). This pellet was resuspended in 60 mL of Milli-Q water and gently stirred for 20 min at room temperature before centrifugation at 5000 x *g* for 20 min (SS-34 rotor; Sorvall). The supernatant containing pili was precipitated once more in the presence of 5% (w/v) PEG 6000, 0.5 M NaCl as previously, before a final centrifugation step at 27'000 x *g* for 30 min (SS-34 rotor; Sorvall). Pili were resuspended in 500 μL of buffer A (20 mM Tris-HCl [pH 7.5], 150 mM NaCl) before being layered onto a pre-formed CsCl step gradient (1.1-1.4 g/cm^3) and centrifuged at 200'000 x *g* for 17 h (SW 60 Ti rotor; Beckman Coulter). The pili containing band was carefully removed and dialysed against buffer A. Once fully dialysed, the pili containing solution was applied to a 15%-60% (w/v) sucrose density gradient and centrifuged at 114'000 x *g* for 12 h (SW 40 Ti rotor; Beckman Coulter). Fractions from the sucrose density gradient were analysed by SDS-PAGE using 4%-12% NuPage gradient gels (Life Technologies) and pili containing fractions were pooled. The final sample was dialysed against buffer A and the identity of type 1 chaperone-usher pili was confirmed by the identification of FimA, FimF, FimG and FimH by mass spectrometry. All purification and centrifugation steps were carried out at 4°C unless stated otherwise.

Production and Purification of FimA Monomers

Mature FimA without its natural signal sequence (159 residues) was produced at 37°C in the cytoplasm of *E. coli* BL21 (DE3) in the form of cytoplasmic inclusion bodies using the T7 expression plasmid described previously (Puorger et al., 2011). FimA production was induced at an OD_{600} of ~0.7 with IPTG (1 mM final concentration) and cells were grown further for 5 h. Cells were harvested by centrifugation, suspended 100 mM Tris-HCl [pH 8.0], 1 mM PMSF, 2 mM $MgCl_2$, 10 μg/mL DNaseI, supplemented with cOmplete™ mini EDTA-free protease inhibitor cocktail (Roche, 1 tablet/100 mL) and lysed (Microfluidics cell cracker). The lysate was mixed with 0.8 volumes of 60 mM EDTA-NaOH [pH 7.0], 1.5 M NaCl, 6% (v/v) Triton X-100, incubated for 30 min at 4°C and centrifuged (48'000 x *g*, 4°C). The inclusion body pellet was washed extensively with 100 mM Tris-HCl [pH 8.0], 20 mM EDTA and solubilized with 6 M GdmCl, 50 mM Tris-HCl [pH 8.0], 1 mM EDTA, 50 mM DTT. After incubation for 2 h at room temperature, insoluble material was removed by centrifugation (100'000 x *g*, 30 min). DTT and EDTA in the supernatant containing unfolded, reduced FimA were removed on a Sephadex G 25 desalting column in the presence of 6 M GdmCl. The intramolecular disulfide bond in FimA was formed

via Cu^{2+}-catalyzed air oxidation in 6 M GdmCl, 50 mM Tris-HCl [pH 8.0], 0.1 μM $CuCl_2$ at low FimA concentration (5 μM) to prevent formation of intermolecular disulfides (room temperature, 18 h). The absence of free thiols at the end of the reaction was verified with Ellman's assay (Ellman, 1959). The solution was then concentrated by cross-flow filtration (10 kDa Hydrosart® membrane cassettes, Sartorius) and disulfide-intact FimA was refolded by dilution with 20 mM H_3PO_4-NaOH [pH 7.0], 150 mM NaCl, 1 mM EDTA and dialysis against 20 mM H_3PO_4-NaOH [pH 7.0], 150 mM NaCl (final FimA concentration during refolding: 50 μM). The refolded FimA monomer was then purified by size exclusion chromatography on Superdex 75 in 20 mM MOPS-NaOH [pH 7.0], 150 mM NaCl and stored in 5 mM MOPS-NaOH [pH 7.0] The final yield of the FimA monomer was 30 mg per liter of bacterial culture. The concentration of the FimA monomer was determined via its specific absorbance at 280 nm (ε_{280} = 2680 M^{-1} cm^{-1}). Electrospray mass spectrometry showed that the N-terminal methionine introduced for cytoplasmic expression of FimA had been cleaved off quantitatively (measured: 15826.0 Da; calculated: 15827.4 Da).

In Vitro Assembly of Type 1 Pilus Rods

The FimA monomer was dialyzed against 20 mM NH_4HCO_3, lyophilized and dissolved in 20 mM acetic acid-NaOH [pH 5.0], 150 mM NaCl. After removal of insoluble material by centrifugation (25'000 x *g*, 5 min), the *in vitro* assembly of FimA monomers to pilus rods at a total FimA monomer concentration of 50 μM in 20 mM acetic acid-NaOH [pH 5.0], 150 mM NaCl occurred during incubation for 7 days. The assembled pilus rods were then pelleted by centrifugation (186'000 x *g*, 10 min, 4°C), washed with 20 mM acetic acid-NaOH [pH 5.0], 150 mM NaCl and finally suspended in the same buffer to a final FimA (monomer) concentration of 400 μM.

In Vivo Pilus Assembly for Stability Studies

E. coli W3110Δ*fimA* transformed with the FimC/FimA coexpression plasmid pCG1-AC (*tet* promoter control) was grown at 37°C in 2YT medium supplemented with ampicillin (100 mg/L) and anhydrotetracycline (12.5 ng/ml). Cells were grown for 18 h, harvested by centrifugation and suspended in 20 mM Tris-HCl [pH 8.0] (5 mL per gram of cells) using a DIAX 600 disperser (Heidolph) set to 8'000 rpm. The suspension was incubated at 90°C for 20 min and centrifuged (10 min, 20'000 x *g*, 20°C). Pili were precipitated by addition of $MgCl_2$ (final concentration: 0.1 M) and incubation for 1 h on ice, harvested by centrifugation (15 min, 48'000 x *g*, 20°C), and washed twice with 20 mM Tris-HCl [pH 8.0], 0.1 M $MgCl_2$, 1 % (w/v) SDS (removal of supernatant after centrifugation for 15 min at 48'000 x *g*, 20°C, respectively). After a third washing step (same conditions without SDS) the pili were suspended in 20 mM Tris-HCl [pH 8.0] and dialyzed against 20 mM H_3PO_4-NaOH [pH 7.0], 50 mM NaCl (membrane with 300 kDa molecular weight cutoff, Spectrum Laboratories). Insoluble material was removed by centrifugation (15 min, 48'000 x *g*, 20°C). The pilus concentration in the supernatant was determined via the specific absorbance of FimA at 280 nm and corrected for light scattering as described (Colón, 1999). The final yields of purified pili were 15–20 mg per liter of bacterial culture, and the pili were stored at 4°C. For recording of dissociation/unfolding kinetics, the pili were pelleted by centrifugation (186'000 x *g*, 10 min, 4°C), washed with 20 mM acetic acid-NaOH [pH 5.0], 150 mM NaCl and finally suspended in the same buffer to a final FimA (monomer) concentration of 400 μM.

Dissociation and Unfolding Kinetics

Preparations of pilus rods (400 μM FimA (monomer) in 20 mM acetic acid-NaOH [pH 5.0], 150 mM NaCl) were diluted 1:40 (final FimA concentration: 10 μM; manual mixing) with 20 mM H_3PO_4-NaOH [pH 2.1] containing different GdmCl concentrations, and the kinetics of dissociation/unfolding were recorded at 25°C via the decrease in the far-UV circular dichroism (CD) signal at 230 nm using JASCO J-715 Spectropolarimeter. Final GdmCl concentrations were verified via their refractive index (Nozaki, 1972). All dissociation/unfolding kinetics were consistent with a single first-order reaction and were fitted according to equation

$$S = S_\infty + (S_0 - S_\infty)\cdot e^{-kt}$$

where S, S_0 and S_∞ are the measured, initial and final CD signals, k is the rate constant of dissociation and unfolding, and t is reaction time. The logarithms of the determined rate constants were then plotted against GdmCl concentration and fitted linearly in the case of the *in vitro* assembled pili. The nonlinear dependence of ln k on GdmCl concentration observed for pili assembled *in vivo* was tentatively fitted to the model of a high-energy on-pathway intermediate of folding/unfolding (A. Bachmann and Kiefhaber, 2001).

Cryo-EM Sample Preparation and Data Collection

A 3 μL sample of type 1 chaperone-usher pili was applied to a glow-discharged Quantifoil 1.2/1.3 400 mesh grid (Agar Scientific) and incubated for 30 s before being blotted and plunged into liquid ethane using a Vitrobot plunge-freezing device (FEI). The data were collected on a Tecnai G^2 Polara microscope (FEI) operated at 300 kV equipped with a K2 Summit direct electron detector (Gatan) operated in counting mode, placed at the end of a Quantum energy filter operated with a slit width of 20 eV, with a 1.13 Å pixel size and a defocus range of -0.5 to -3.5 μm. A total dose of 100 electrons/$Å^2$ was applied and fractionated equally among 59 frames to allow for dose weighting.

Cryo-EM Image Processing and Reconstruction

Whole-image drift correction to align the 59 movie frames of each micrograph was carried out using MOTIONCOR2 (Zheng et al., 2017) and the contrast transfer function (CTF) parameters of the corrected micrographs were estimated using GCTF (Zhang, 2016). The implementation for the reconstruction of helical assemblies in the program RELION-2.0 (He and Scheres, 2017;

Scheres, 2012) was used for image processing and reconstruction. Filaments were manually picked from 177 selected micrographs and a total of 115’545 segments were extracted with a box size of 240 pixels. After 2D and 3D classification steps, a total of 115’510 segments were used for 3D refinement. A solid cylinder with a diameter of 100 Å, low-pass filtered to 30 Å, was used as the starting model for 3D reconstruction. Several narrow search ranges for the helical parameters of twist and rise were tested during 3D classification encompassing a total range of 100-116° for twist and 6.8-8.2 Å for rise, which included the previously reported twist and rise values of Hahn et al. (2002) and Habenstein et al. (2015). However, this process identified 3D classes displaying high-resolution structural features only when the ranges encompassed a twist of 115° and a rise of 8.0 Å, indicating that these were the correct helical parameters. Therefore, the final helical parameters were refined using a search range of 114° to 116° for the twist and 7.9 Å to 8.1 Å for the rise. No segments were discarded after 3D classification, as all three classes refined with highly similar helical parameters. Please refer to Table S1 for details of the helical parameters after 3D classification and refinement. During the post-processing step in RELION-2.0, a soft mask with a raised cosine edge 7 pixels wide was employed yielding a final map with a global resolution of 4.2 Å as assessed by the gold standard FSC procedure implemented in RELION-2.0 (FSC=0.143) (Rosenthal and Henderson, 2003), consistent with strand separation and clear density for bulky side chains.

Model Building, Refinement, Structure Analysis

The FimA pilin (PDB ID: 2JTY (Puorger et al., 2011)) structure encompassing residues 20-159 was docked into the map using Chimera (Pettersen et al., 2004). The donor-strand was initially modelled with residues 172-182 (representing Nte residues 7-17) from the self-complemented donor strand of the same FimA pilin structure (PDB ID: 2JTY). The Nte residues were renumbered and missing residues (2-6 and 18-19) were manually built using *Coot* (Emsley et al., 2010). The final model encompasses residues 2-158 of FimA, missing one residue from both the N and C terminus due to poorly defined electron density. A model containing six molecules of FimA was refined using several cycles of PHENIX (Real Space Refine) (Adams et al., 2010). Manual adjustment in *Coot* and structure idealisation in REFMAC5 (Vagin et al., 2004) was performed to improve the geometry of the model between cycles of real space refinement. Knowledge of the FimA structure from previous NMR and crystallography studies were used to guide building and refinement (Crespo et al., 2012; Puorger et al., 2011; Walczak et al., 2014). However, in order to obtain an unbiased view of secondary structure element boundaries, the DSSP server (Joosten et al., 2010; Kabsch and Sander, 1983) was used in combination with careful manual examination of the model in *Coot* to delineate the final secondary structure element boundaries enforced during real space refinement. Final validation of the model was performed using MOLPROBITY (Chen et al., 2010) and the wwPDB validation Service. A Fourier Shell Correlation (FSC) curve was calculated to assess the agreement between the map and the model and to avoid potential overfitting. The pairwise alignment function of the DALI server was used to calculate RMSD values for the alignment of two structures across a range of Cα atoms (Holm and Laakso, 2016). The CoCoMaps Tool was used to analyse the interfaces between FimA or PapA pilins in the quaternary structure of their respective pili (Vangone et al., 2011).

QUANTIFICATION AND STATISTICAL ANALYSIS

Quantification and statistical analyses employed in this publication pertain to the analysis on electron microscopy data and the determination of structures by electron microscopy, which are integral parts of existing algorithms and software used.

DATA AND SOFTWARE AVAILABILITY

The accession number for the EM map reported in this paper is EMD-3809. The accession number for the model coordinates deposited in the PDB is 5OH0.

Structure, Volume *25*

Supplemental Information

The Cryoelectron Microscopy Structure of the Type 1 Chaperone-Usher Pilus Rod

Manuela K. Hospenthal, Dawid Zyla, Tiago R.D. Costa, Adam Redzej, Christoph Giese, James Lillington, Rudi Glockshuber, and Gabriel Waksman

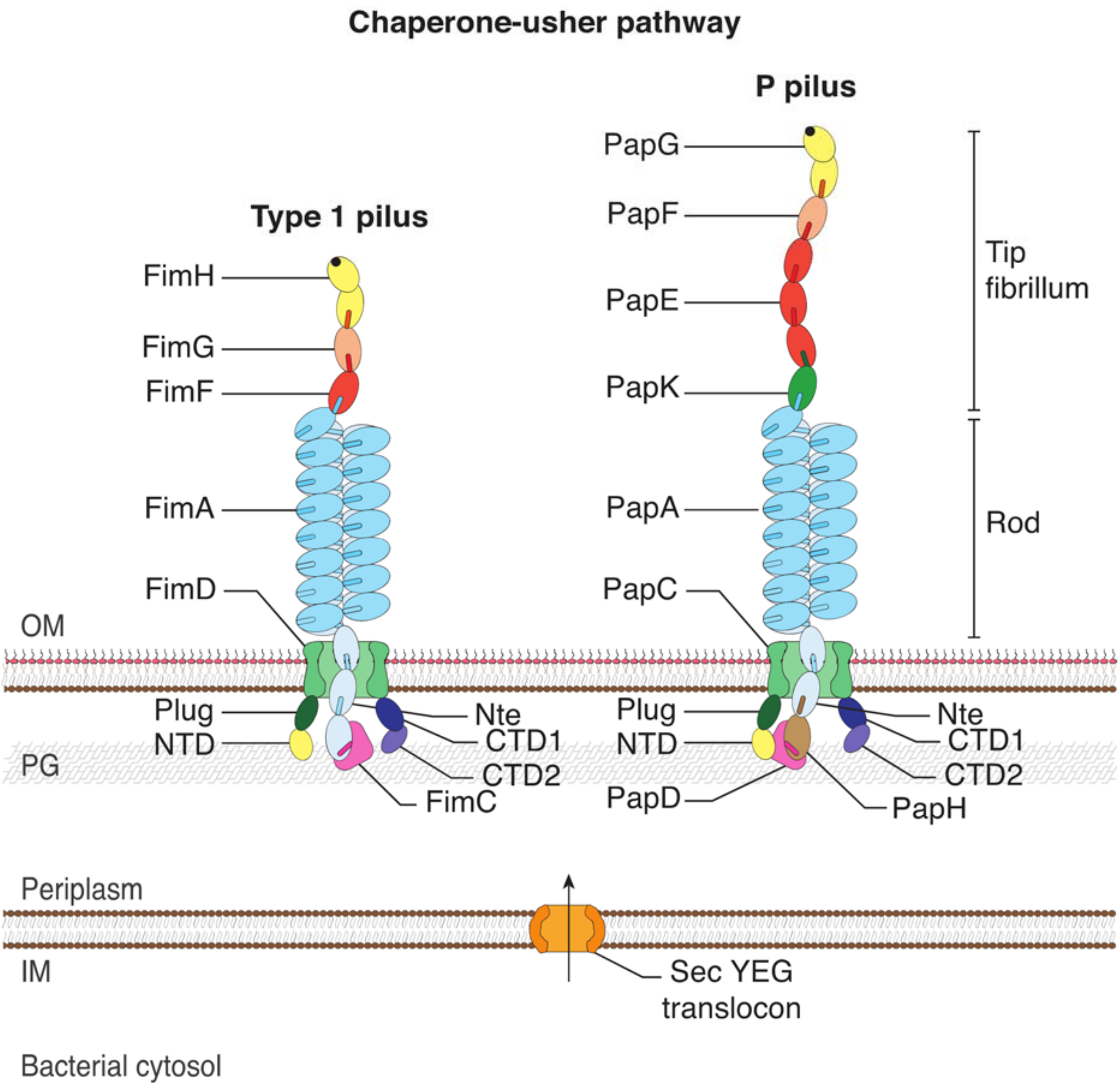
Chaperone-usher pathway
Type 1 pilus
P pilus
FimH
FimG
FimF
FimA
FimD
OM
Plug
NTD
PG
Nte
CTD1
CTD2
FimC
PapG
PapF
PapE
PapK
PapA
PapC
Plug
NTD
PapD
Nte
CTD1
CTD2
PapH
Tip
fibrillum
Rod
Periplasm
IM
Sec YEG
translocon
Bacterial cytosol

Figure S1: The Chaperone-Usher Pathway. Related to Figure 1, 2, 3, 4 and 5.

Pilin subunits are transported across the bacterial inner membrane (IM) via the Sec YEG translocon where they are received by a dedicated periplasmic chaperone (FimC, type 1 pili; PapD, P pili). The chaperone helps to fold and stabilise the pilin subunits and shuttles them to the outer membrane (OM)-embedded usher, where they are polymerised into a growing pilus. The usher is composed of a 24-stranded β-barrel pore, a periplasmic N-terminal domain (NTD), two periplasmic C-terminal domains (CTD1 and CTD2) and a plug domain. Once assembled, chaperone-usher pili are organised into two subassemblies: the tip fibrillum and a 1-2 μm long rod. The tip fibrillum is assembled first and requires the sequential addition of a single subunit of FimH, FimG and FimF (type 1 pili) or one subunit of PapG and PapF, 5-10 subunits of PapE and one PapK subunit (P pili). FimH and PapG are the adhesins composed of a C-terminal pilin domain and an N-terminal lectin domain, which is responsible for host-cell receptor interaction. The rod section is composed of ~1000 subunits of FimA (type 1 pili) or PapA (P pili).

a

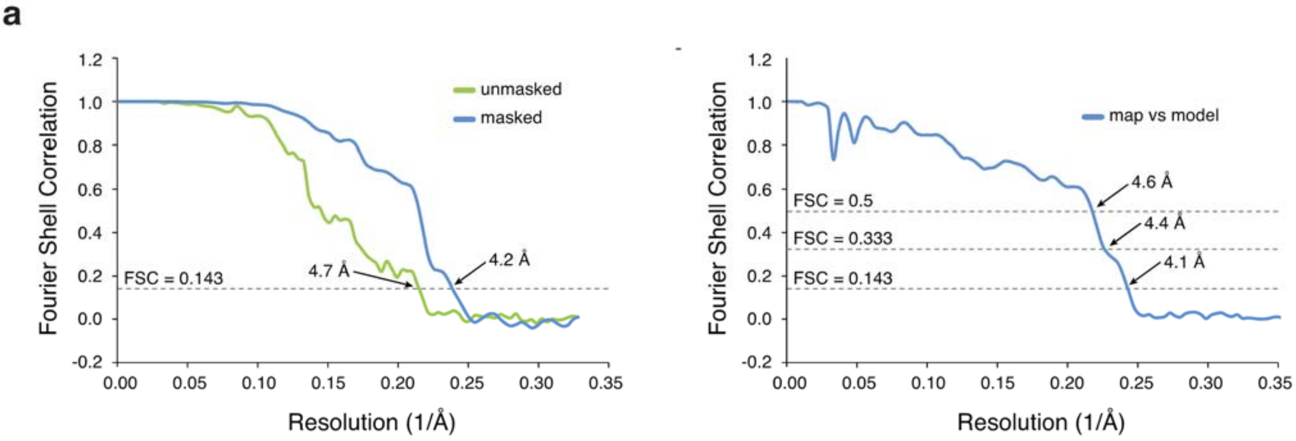

b

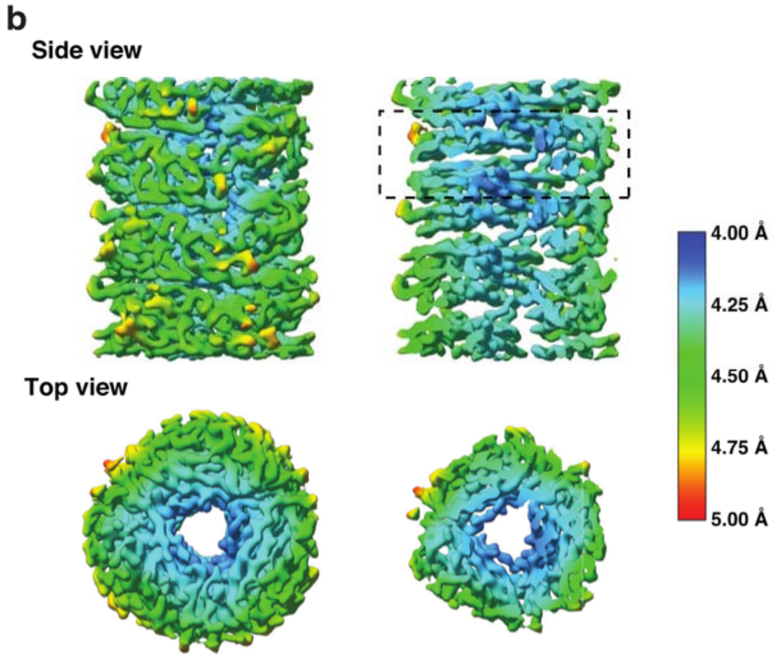

c

Molprobity score	1.12
All-atom clashscore	3.32
Ramachandran plot:	
Outliers (%)	0.00
Allowed (%)	1.93
Favoured (%)	98.07
RMS deviations (angles)(°)	0.62
RMS deviations (bonds)(Å)	0.00
Bad rotamers (%)	0.00

Figure S2: Resolution Analysis, Model Building and Refinement. Related to Figure 1.

a Left, Fourier Shell Correlation (FSC) curves calculated for unmasked (green) and masked (blue) reconstructions. Right, FSC curve calculated for the map versus model. Resolutions at the indicated FSC cut-offs are indicated on the graph. **b** Analysis of local resolution indicating that the luminal side of the pilus is better resolved than the exterior. A side view (upper panels) and bottom view (lower panels) of the map surface coloured according to the local resolution (indicated by the scale bar). The left panels show the whole map, whereas the right panels show a sliced view revealing more detail in the pilus interior. The dashed box on the sliced side view (upper right) represents the portion of the map included in the sliced top view (lower right). **c** Model validation statistics.

a

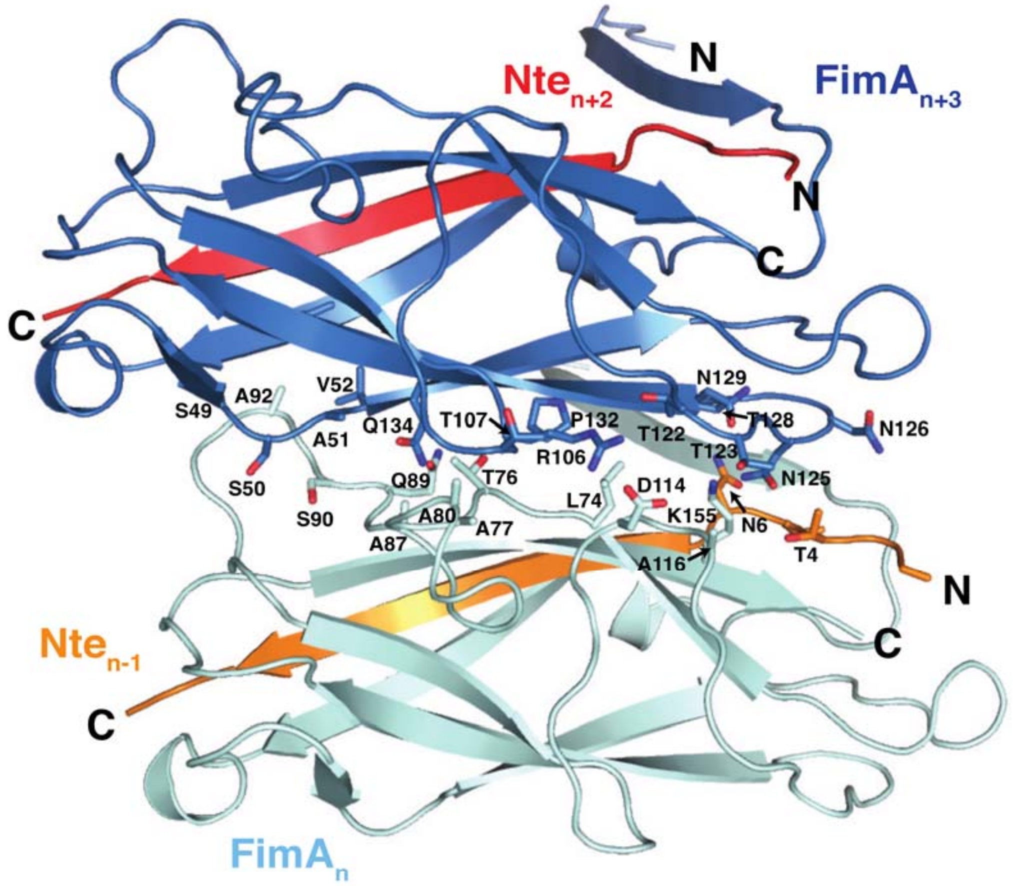

b

Stacking interface analysis

	Type 1	Type 1 (- DS)	P	P (- DS)
**Total buried surface area (Å)**	1616.2	1430.2	1453.0	1150.3

Figure S3: The Main Stacking Interface. Related to Figure 2.

a Zoomed-in view of the stacking interface in the same orientation as in Figure 2c showing participating side chains. The two FimA subunits are coloured in light and dark blue and are shown in ribbon representation. Donor-strands are coloured orange and red. Side chains are shown in stick representation with oxygens coloured red and nitrogens coloured blue. **b** Table displaying the total buried surface areas created by the n and n+3 (stacking) interface in type 1 and P pili. Values were calculated for the stacking interface with and without the donor strand (DS).

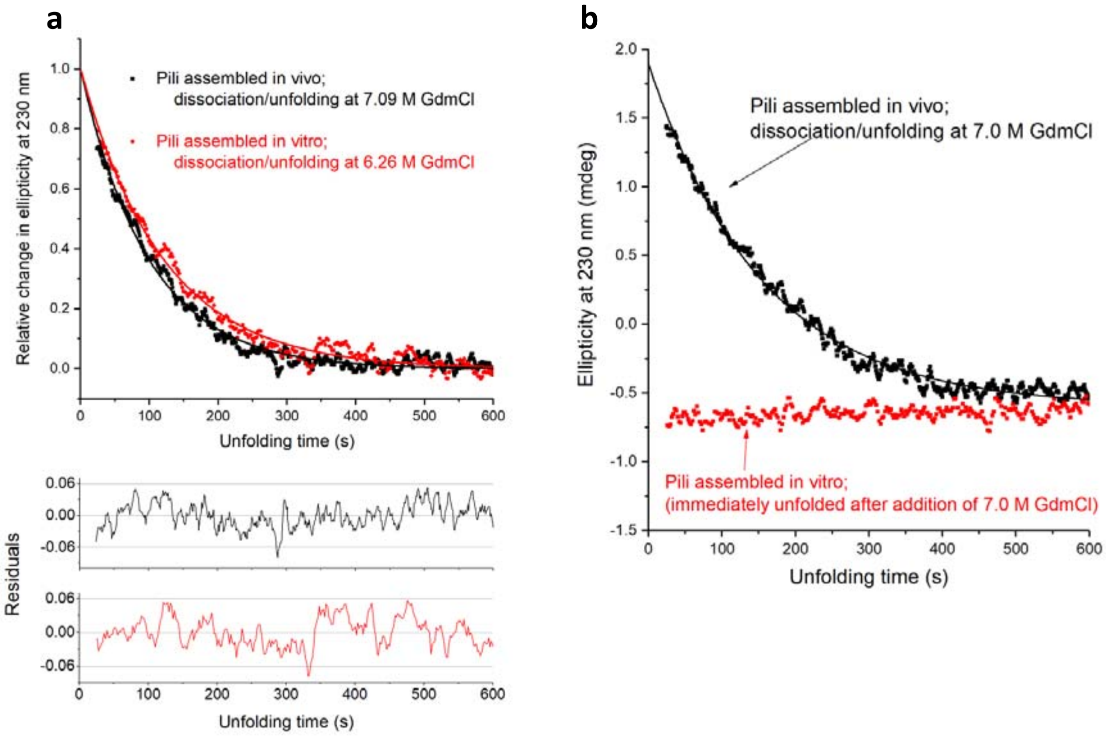

Figure S4: Unfolding/Dissociation Traces of Type 1 Pilus Rods Assembled *in vivo* and *in vitro.* Related to Figure 5.

a Unfolding traces recorded of type 1 pilus rods assembled *in vivo* (black symbols) and *in vitro* (red symbols) via the decrease in the far-UV CD signal at 230 nm at pH 2.1 and 25°C of pili assembled *in vivo* at 7.09 M GdmCl and pili assembled from FimA monomers *in vitro* at 6.26 M GdmCl. The data were fitted to a single first-order reaction (solid lines). The residuals at the bottom show that there are no systematic deviations form first-order kinetics. **b** Dissociation/unfolding at pH 2.1 and 7.0 M GdmCl. While pili assembled *in vivo* slowly dissociate with a half-life of 107±2 s, pili assembled *in vitro* already dissociate and unfold within the dead time of manual mixing under these conditions.

SUPPLEMENTAL TABLES

Table S1 Overview of the Refinement of Helical Parameters during EM Processing. Related to Experimental Procedures.

	**Twist (°)**	**Rise (Å)**
**After 3D classification**		
Class 1	115.202	8.02695
Class 2	114.999	8.00031
Class 3	114.984	8.01445
**After 3D refinement**		
Half 1	114.993	8.01008
Half 2	114.991	8.01367
Average	114.992	8.01188
